# Multiomics analysis reveals that hepatocyte nuclear factor 1β regulates axon guidance genes in the developing mouse kidney

**DOI:** 10.1038/s41598-022-22327-5

**Published:** 2022-10-20

**Authors:** Annie Shao, Micah D. Gearhart, Siu Chiu Chan, Zhen Miao, Katalin Susztak, Peter Igarashi

**Affiliations:** 1 Department of Medicine, University of Minnesota Medical School, 420 Delaware Street SE, MMC 194, Minneapolis, MN 55455 USA; 2 Molecular, Cellular, Developmental Biology and Genetics Graduate Program, University of Minnesota, Minneapolis, MN USA; 3 Department of Genetics, Cell Biology and Development, University of Minnesota, Minneapolis, MN USA; 4 Renal, Electrolyte, and Hypertension Division, Department of Medicine, University of Pennsylvania, Perelman School of Medicine, Philadelphia, PA USA

**Keywords:** Kidney, Transcription, Organogenesis, Cell signalling, Cell biology, Developmental biology, Genomics, Paediatric kidney disease, Polycystic kidney disease

## Abstract

The transcription factor hepatocyte nuclear factor 1β (HNF-1β) is essential for normal development of the kidney and other epithelial organs. In the developing mouse kidney, HNF-1β is required for the differentiation and patterning of immature nephrons and branching morphogenesis of the ureteric bud (UB). Here, we used ChIP-sequencing (ChIP-seq) and RNA sequencing (RNA-seq) to identify genes that are regulated by HNF-1β in embryonic mouse kidneys. ChIP-seq revealed that HNF-1β binds to 8284 sites in chromatin from E14.5 mouse kidneys. Comparison with previous ATAC-seq and histone modification studies showed that HNF-1β binding peaks colocalized with open chromatin and epigenetic marks of transcriptional activation (H3K27 acetylation, H3K4 trimethylation, H3K4 monomethylation), indicating that the binding sites were functional. To investigate the relationship between HNF-1β binding and HNF-1β-dependent gene regulation, RNA-seq was performed on UB cells purified from wild-type and HNF-1β mutant embryonic kidneys. A total of 1632 genes showed reduced expression in HNF-1β-deficient UB cells, and 485 genes contained nearby HNF-1β binding sites indicating that they were directly activated by HNF-1β. Conversely, HNF-1β directly repressed the expression of 526 genes in the UB. Comparison with snATAC-seq analysis of UB-derived cells showed that both HNF-1β-dependent activation and repression correlated with chromatin accessibility. Pathway analysis revealed that HNF-1β binds near 68 axon guidance genes in the developing kidney. RNA-seq analysis showed that *Nrp1*, *Sema3c*, *Sema3d*, *Sema6a*, and *Slit2* were activated by HNF-1β, whereas *Efna1*, *Epha3*, *Epha4*, *Epha7*, *Ntn4*, *Plxna2*, *Sema3a*, *Sema4b*, *Slit3*, *Srgap1*, *Unc5c* and *Unc5d* were repressed by HNF-1β. RNAscope in situ hybridization showed that *Nrp1*, *Sema3c*, *Sema3d*, *Sema6a*, and *Slit2* were expressed in wild-type UB and were dysregulated in HNF-1β mutant UB. These studies show that HNF-1β directly regulates the expression of multiple axon guidance genes in the developing mouse kidney. Dysregulation of axon guidance genes may underlie kidney defects in HNF-1β mutant mice.

## Introduction

The kidneys are paired epithelial organs that are embryologically derived from intermediate mesoderm^1^. The definitive kidney in mammals, the metanephros, originates from two distinct primordia: the ureteric bud (UB) is an outgrowth from the posterior Wolffian duct that elongates and undergoes branching morphogenesis to produce the renal collecting system. The adjacent metanephric mesenchyme (MM) contains stem cells that are the progenitors of nephrons, the functional units of the kidney. Kidney development is driven by reciprocal interactions between the UB and MM. The MM secretes factors such as glial cell line-derived neurotrophic factor (GDNF) that signal to receptors located on the UB and induce UB elongation and branching^1,2^. Conversely, the tips of the UB secrete factors, such as Wnt9b, that induce the adjacent MM to undergo mesenchymal-epithelial transition forming immature nephrons^3^. With further development, the nephrons become patterned into proximal and distal segments and fuse with the collecting ducts forming a continuous lumen.

Hepatocyte nuclear factor 1β (HNF-1β) is a POU-homeodomain transcription factor that regulates tissue-specific gene expression in epithelial organs, including the kidney^4^. HNF-1β recognizes the consensus sequence 5′-GGTTAATNATTAAC-3′ that is often found in promoter or enhancer regions of HNF-1β target genes^5,6^. HNF-1β activates gene transcription by recruiting the histone acetyltransferases CREB-binding protein (CBP) and P300/CBP-associated factor (PCAF)^7,8^. HNF-1β can also repress gene transcription by recruiting histone deactylase-1^7^. HNF-1β is expressed in all tubular epithelial cells in the kidney. In the developing mouse kidney, HNF-1β is detected in the Wolffian duct at embryonic day (E) 9.5 and is subsequently expressed in the branching UB^4^. HNF-1β is absent in the MM, but its expression is induced coincident with the formation of epithelia^9,10^. Expression of HNF-1β is maintained in adult nephrons and collecting ducts^4,9^. Gene targeting studies have revealed that HNF-1β is required at multiple steps of kidney development. Inactivation of HNF-1β in the UB significantly reduces branching morphogenesis and the induction of new nephrons^11^. Ablation of HNF-1β in nephron progenitors in the MM impairs nephron differentiation and inhibits the formation of the proximal and distal tubules and loops of Henle^10^.

Heterozygous mutations of *HNF1B* in humans are among the most common monogenic causes of congenital kidney abnormalities^12^. Mutations of *HNF1B* account for 10–30% of cases of congenital anomalies of the kidney and urinary tract (CAKUT)^13^. Over 100 pathogenic mutations in *HNF1B* have been reported^14^, including whole-gene deletions, point mutations and small insertions^12,15,16^. *HNF1B* mutations have an autosomal dominant inheritance, though there are also sporadic mutations^13^. Renal manifestations of *HNF1B* mutations include kidney agenesis, hypoplasia, dysplasia, duplicated ureters, and multicystic dysplastic kidneys^17,18^. Extrarenal phenotypes associated with HNF-1β-related CAKUT include hypomagnesemia^19^, diabetes^20^, female genital tract malformations^21^, and neurodevelopmental disorders^12,22^. To gain mechanistic insights into the functions of HNF-1β in kidney development, we conducted a genomewide analysis of HNF-1β gene targets in the embryonic mouse kidney. We specifically focused on genes expressed in the UB that may underlie branching defects in HNF-1β mutant animals.

## Results

### HNF-1β primarily binds gene promoters and intragenic regions in the developing kidney

**Figure 1:**
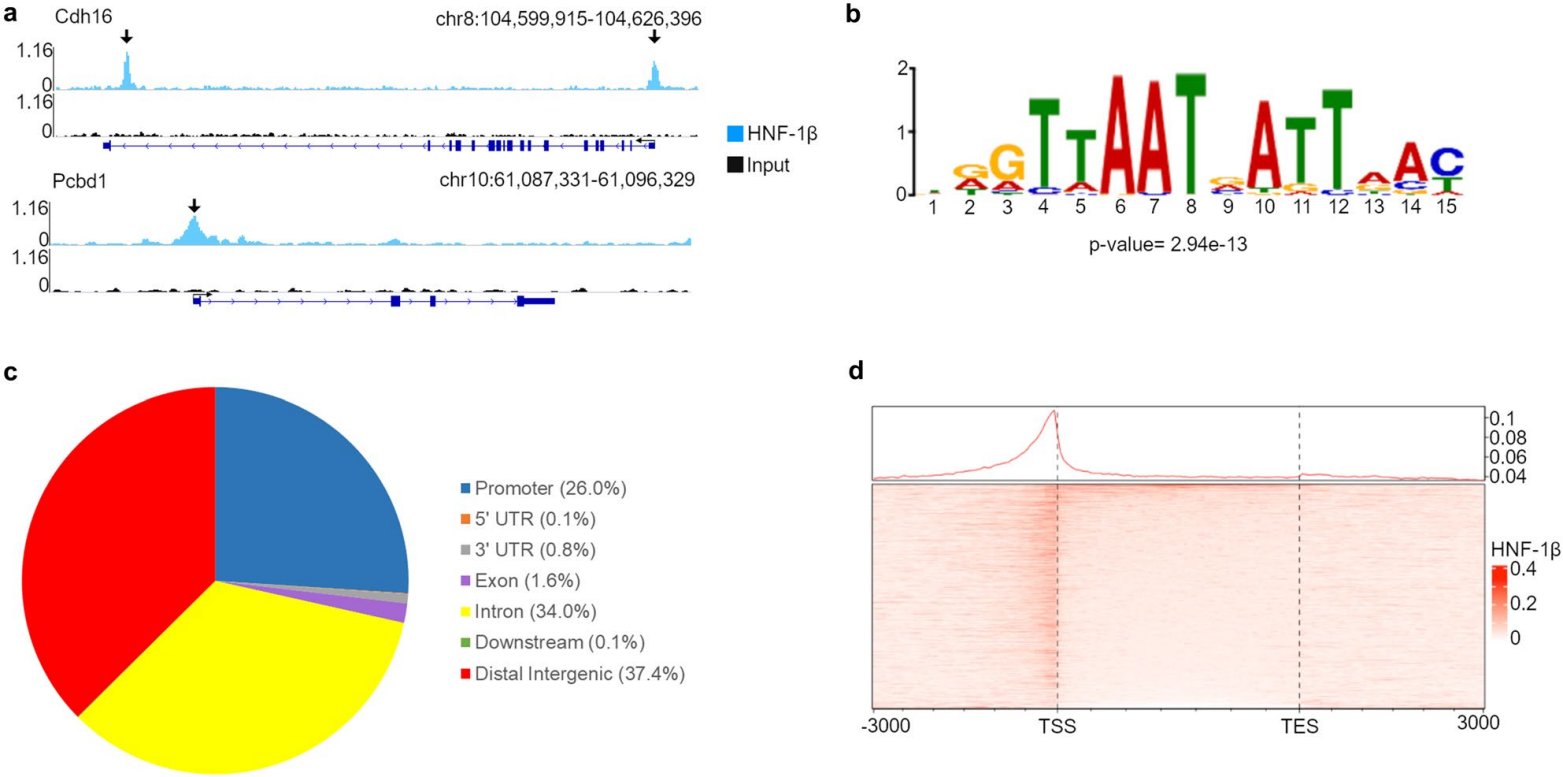
Identification of HNF-1β binding sites in developing mouse kidneys. (**a**) Representative ChIP-seq peaks in chromatin from E14.5 mouse kidneys. The light blue track shows the HNF-1β immunoprecipitation, and the black track shows the input sample. The gene structures are shown below (dark blue). Thin lines indicate introns, thick bars indicate exons, and arrows indicate the direction of transcription. The transcription start site is indicated by the bent arrow. Vertical arrows indicate HNF-1β binding peaks in the promoter and 17th intron of *Cdh16* (upper panel) and the promoter of *Pcbd1* (lower panel). (**b**) De novo motif analysis of HNF-1β binding peaks. (**c**) Pie chart summarizing the location of HNF-1β binding sites in chromatin from E14.5 mouse kidneys. The promoter region was defined as 3 kb upstream from the transcription start site (TSS), downstream was defined as < 300 bp downstream from the transcription end site (TES), and distal intergenic was defined as > 300 bp downstream of the TES or > 3 kb upstream from the TSS. (**d**) Heatmap showing HNF-1β ChIP-seq enrichment in protein-coding genes from the TSS to the TES and including 3000 bp of 5′ and 3′ flanking sequence.

To identify HNF-1β binding sites in the developing kidney, we performed chromatin immunoprecipitation followed by high-throughput sequencing (ChIP-seq). Kidneys (metanephroi) were dissected from wild-type embryos at E14.5 and pooled. Kidneys from two independent pooled litters were analyzed separately (two biological replicates). Chromatin was extracted from the kidneys, cross-linked, and immunoprecipitated with an anti-HNF-1β antibody. Cross-linking was reversed, and DNA libraries were generated and sequenced. Read depth and ChIP-seq quality measures can be found in Supplementary Table S1. Mapping to the mouse genome (mm10) identified 8284 HNF-1β binding sites that were common in the two biological replicates. Peaks were found at known HNF-1β target genes, including *Cdh16* and *Pcbd1* (Fig. 1a). The locations of the peaks matched the locations of previously identified HNF-1β binding sites^23,24^, which validated the ChIP-seq methodology in embryonic kidneys. De novo motif analysis was performed on the top 500 HNF-1β peaks after filtering out low-complexity regions and simple repeats (Fig. 1b). The HNF-1β consensus sequence was highly represented in these peaks (p = 2.9 × 10^–13^).

Of the HNF-1β binding sites, 26% were located in gene promoters, which were defined as 3 kb upstream from the transcription start site (TSS); 37% were intragenic, defined as the 5′-UTR, 3′-UTR, introns, exons, and 300 bp downstream from the transcription end site (TES); and 37% were located in distal intergenic regions, defined as > 300 bp downstream of the TES or > 3 kb upstream from the TSS (Fig. 1c). HNF-1β binding was visualized across the gene body by aligning the TSS and TES of each protein-coding gene and creating a heatmap of HNF-1β ChIP-seq reads. HNF-1β showed the highest binding in promoters immediately upstream from the TSS, and lower levels of binding were observed within the body of genes. Another small peak of enrichment was found immediately downstream from the TES (Fig. 1d).

Of the total HNF-1β binding sites, 5068 sites were located within or in closest proximity to protein-coding genes. Most of these sites were located in gene promoters (30%) or were intragenic (51%). Only 19% were distal intergenic. A total of 982 sites mapped to long non-coding RNAs, of which 17% were in the promoter region, 27% were intragenic, and 56% were distal intergenic. A total of 134 sites mapped to microRNAs, of which 29% were in the promoter region and 71% were distal intergenic. The remaining gene classifications of HNF-1β peaks can be found in Supplementary Table S2. Taken together, the HNF-1β consensus sequence highly represented in the E14.5 ChIP-seq, as well as known HNF-1β binding sites. The majority of HNF-1β peaks in protein-coding genes were within the promoter and gene body, whereas most peaks in non-coding genes were located in distal intergenic regions.

### HNF-1β binding sites in the developing kidney colocalize with open chromatin and histone modifications

To determine if the HNF-1β binding sites were functional, we examined the relationship between HNF-1β binding and chromatin accessibility. Transcriptional activation is associated with open chromatin, which can be measured using ATAC-seq (Assay for Transposase Accessible Chromatin)^25^. Moreover, HNF-1β itself functions as a pioneer transcription factor that remains bound to DNA through mitosis and can activate transcription by promoting histone modifications that result in increased chromatin accessibility^26^. To determine whether HNF-1β binding in embryonic mouse kidneys is associated with increased chromatin accessibility, we compared the locations of HNF-1β ChIP-seq peaks and ATAC-seq peaks. Here, we took advantage of an existing ATAC-seq dataset from the ENCODE database^27,28^ obtained from wild-type E14.5 mouse kidney^29^, the same tissue source used for HNF-1β ChIP-seq. We identified 2263 HNF-1β ChIP-seq peaks that colocalized with ATAC-seq peaks, which represented 27% of the total HNF-1β peaks. To validate this colocalization with HNF-1β activity, binding sites at known HNF-1β targets were examined. The HNF-1β binding sites at the promoters of *Cdh16* and *Pcbd1* overlapped with open chromatin (Fig. S1). The association between HNF-1β binding and ATAC-seq peaks was statistically significant (p < 2 × 10^–16^, odds ratio = 16.5, hypergeometric test).

**Figure 2:**
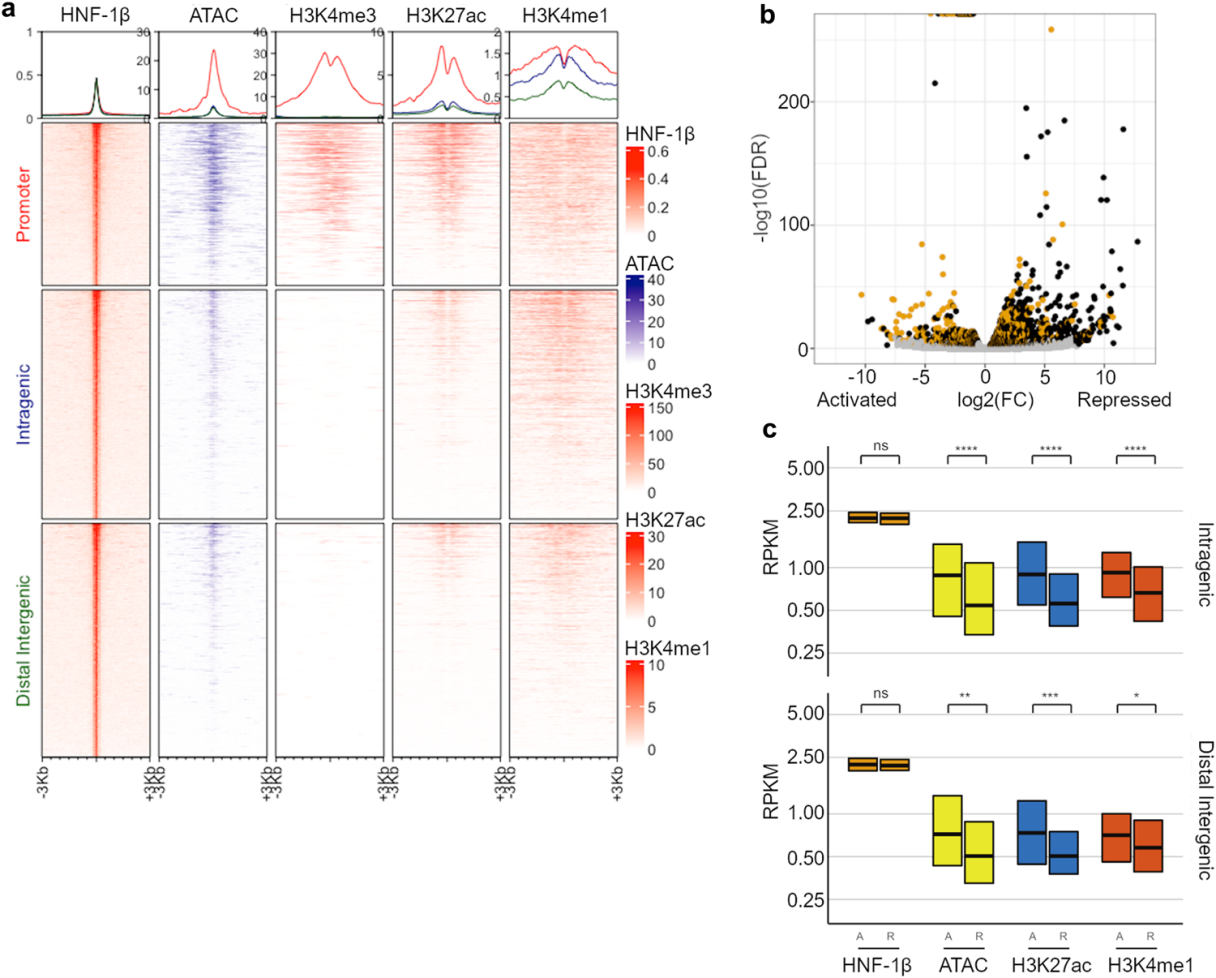
Genome-wide analysis of open chromatin and histone modifications at HNF-1β binding sites in developing mouse kidneys. (**a**) Enrichment of ATAC-seq, H3K4 trimethylation (H3K4me3), H3K27 acetylation (H3K27ac), and H3K4 monomethylation (H3K4me1) at HNF-1β binding sites in chromatin from E14.5 kidney. Enrichment is shown in the promoter (upper panel), intragenic (middle panel), and distal intergenic regions (lower panel). Peaks are centered on HNF-1β binding sites (left panels). Top panel shows total binding in the promoter (red line), intragenic (blue line), and distal intergenic regions (green line). (**b**) Volcano plot of RNA-seq results showing genes that are activated or repressed in purified UB cells from heterozygous control E14.5 kidneys compared to HNF-1β mutant kidneys. Gold circles indicate differentially expressed genes that contain nearby HNF-1β binding peaks. (**c**) Comparison of HNF-1β binding, ATAC-seq signal, H3K27ac, and H3K4me1 in genes that are directly activated (A) or repressed (R) by HNF-1β. Binding in intragenic (upper panel) and distal intergenic regions (lower panel) is shown. Normalized enrichment values are shown as reads per kilobase of transcript (RPKM). Boxplots indicate the median and interquartile range. Asterisks indicate statistically significant differences using the Wilcoxon test (*p < 0.05; **p ≤ 0.01; ***p ≤ 0.001; ****p ≤ 0.0001; ns, p ≥ 0.05). Median and mean enrichment values and individual Wilcoxon test results can be found in Supplementary Table S5.

Next, we examined the colocalization of ATAC-seq and HNF-1β peaks in different genomic regions. Chromatin accessibility was greater at HNF-1β binding sites in promoter regions compared to intragenic and distal intergenic regions (Fig. 2a). The HNF-1β enrichment was equal in peaks located in the promoter, intragenic, and distal intergenic regions, so the difference in ATAC-seq signal was not simply due to differences in HNF-1β binding. Instead, these findings may suggest that binding of HNF-1β preferentially increases chromatin accessibility in promoters. One possible mechanism involves histone acetylation. Acetylation of Lys27 on histone H3 (H3K27ac) reduces the electrostatic interaction with DNA and thereby increases chromatin accessibility. Moreover, the C-terminal domain of HNF-1β, which is required for transcriptional activation, has previously been shown to interact with CBP and P/CAF, coactivators that have intrinsic histone acetylase activity^7,8^. Therefore, we tested the relationship between HNF-1β binding and histone acetylation in chromatin from embryonic kidneys. Data from a ChIP-seq analysis of H3K27 acetylation in wild-type E14.5 mouse kidney were downloaded from the ENCODE database^29^. Peaks of H3K27ac enrichment were identified, and the locations were compared with HNF-1β binding peaks. We identified enrichment of H3K27ac at 1970 HNF-1β binding peaks, representing 24% of all HNF-1β binding peaks. HNF-1β binding sites at the promoters of *Cdh16* and *Pcbd1* overlapped with H3K27ac sites (Fig. S1). The association between HNF-1β binding and H3K27 acetylation was statistically significant (p < 2 × 10^–16^, odds ratio = 19.6, hypergeometric test). Similar to the ATAC-seq results, H3K27ac enrichment was higher in promoters compared to intragenic and distal intergenic regions (Fig. 2a).

To further confirm the functional significance of HNF-1β binding, we examined the relationship between HNF-1β binding and histone H3 Lys4 trimethylation (H3K4me3), an epigenetic mark associated with gene activation^30^. Using a similar approach as above, we found enrichment of H3K4me3 at 1002 HNF-1β ChIP-seq peaks in E14.5 mouse kidney. HNF-1β binding sites at the promoters of *Cdh16* and *Pcbd1* overlapped with H3K4me3 sites (Fig. S1). The association between HNF-1β binding and H3K4me3 was statistically significant (p < 2 × 10^–16^, odds ratio = 9.2, hypergeometric test). Enrichment of H3K4me3 was highest at HNF-1β binding peaks in promoter regions and was not detected at HNF-1β binding sites in intragenic and distal intergenic regions (Fig. 2a). Next, we examined the relationship between HNF-1β binding and histone H3 Lys4 monomethylation (H3K4me1), a histone modification associated with primed enhancers^30,31^. We found 1205 H3K4me1 ChIP-seq peaks that overlapped with HNF-1β ChIP-seq peaks. The association between HNF-1β binding and H3K4me1 was statistically significant (p < 2 × 10^–16^, odds ratio = 7.6, hypergeometric test). In contrast to H3K4me3, H3K4me1 colocalized with HNF-1β binding peaks in intragenic and distal intergenic regions in addition to promoter regions (Fig. 2a). Intragenic HNF-1β binding sites at *Gfra1* and *Wnt9b* overlapped with open chromatin, H3K27ac, and H3K4me1, suggesting these are enhancer sites (Fig. S1). The distribution of H3K4me3, H3K27ac, and H3K4me1 at HNF-1β binding sites was bimodal, consistent with the sliding of nucleosomes due to transcription factor binding^32^. The overlap between HNF-1β binding, ATAC-seq, and each histone modification is summarized in Supplementary Fig. S2.

Taken together, HNF-1β binding sites in E14.5 kidneys are associated with regions of open chromatin and activating histone modifications. The colocalization of HNF-1β binding and H3K27ac correlated with ATAC-seq and showed highest enrichment in gene promoters and lower signal in intragenic and distal intergenic regions. Consistent with this finding, colocalization with H3K4me3 was also highest in gene promoters, indicating that HNF-1β binding is associated with promoter activation. In contrast, colocalization with H3K4me3 was absent in intragenic and distal intergenic regions. Instead, HNF-1β peaks in these regions colocalized with H3K4me1, indicating that they corresponded to active enhancers.

### HNF-1β regulates gene expression in purified UB cells

To investigate the relationship between HNF-1β binding and HNF-1β-dependent gene regulation, we performed RNA-seq analysis on UB cells purified from E14.5 mouse kidneys. To determine the dependence on HNF-1β, studies were performed in HNF-1β mutant mice and heterozygous control mice. Using appropriate genetic crosses, we generated Hoxb7/Cre;*Hnf1b*^fl/fl^;tdT (HNF-1β mutant) mice in which Cre recombinase was expressed under the control of the Hoxb7 promoter resulting in inactivation of HNF-1β and activation of a tdTomato reporter gene exclusively in the ureteric bud. Hoxb7/Cre;*Hnf1b*^fl/+^;tdT mice were used as heterozygous controls, as the heterozygotes lack a kidney phenotype. Consistent with previous studies^11^, HNF-1β mutant kidneys were hypoplastic and had reduced UB branching compared to heterozygous control kidneys (Figs. S3 and S4). TdTomato was expressed exclusively in the ureteric bud in both mutant and heterozygous control kidneys, which enabled purification of the UB cells by flow cytometry. Kidneys were dissected from five HNF-1β mutant kidneys and five heterozygous control kidneys at E14.5, and single cell suspensions were prepared by treatment with collagenase. TdTomato-positive UB cells were purified by FACS. Representative analyses are shown in Supplementary Figs. S3 and S4. In total, UB cells accounted for 10.1 ± 1.5% of live cells in wild-type kidneys and 4.6 ± 1.2% of live cells in mutant kidneys. RNA was extracted from the purified UB cells, and cDNA libraries were prepared and sequenced. Each library contained more than 16 million reads (Table S3). All differentially expressed genes had at least 25 mean read counts in one or both genotypes.

UB cells isolated from heterozygous control mice expressed known markers of the ureteric bud, including *Hoxb7*, *Pax2*, *Pax8*, *Ret*, *Gfra1*, and *Sox9*, which validated the preparation of the UB cells (Table S4). UB cells isolated from HNF-1β mutant mice showed a 94% reduction in HNF-1β mRNA levels (Table S4). Comparison of the transcriptomic profiles from HNF-1β mutants and heterozygous controls identified 1632 genes that were more highly expressed in heterozygous control UB cells compared to HNF-1β mutant UB cells (log2fc ≤ − 0.5, FDR < 0.05, negative binomial test with edgeR). We defined these genes as being activated by HNF-1β. Of these genes, 485 (29.7%) contained nearby HNF-1β binding sites, indicating that they were likely directly activated by HNF-1β. As a positive control, we identified genes that had previously been shown to be activated by HNF-1β, including *Wnt9b*, *Cys1*, *Pkhd1*, *Pcsk9*, *Kif12*, and *Cdh16*. Conversely, 2223 genes had significantly lower expression in heterozygous control UB cells compared to HNF-1β mutant UB cells (log2fc ≥ 0.5, FDR < 0.05, negative binomial test with edgeR). We defined these genes as being repressed by HNF-1β. Of these genes, 526 (24.1%) contained nearby HNF-1β binding sites, indicating that they were likely directly repressed by HNF-1β (Fig. 2b). Included in this list were *Socs3* and *Ccdc80*, which we previously showed were repressed by HNF-1β. Fold changes in gene expression and read counts for each sample can be found in Supplementary Table S4. Taken together, these results identified a subset of genes that were directly regulated by HNF-1β in the UB in vivo.

### Histone modifications and increased chromatin accessibility are associated with HNF-1β-dependent gene regulation

Next, we examined whether genes that are activated by HNF-1β have differences in chromatin accessibility or histone modifications compared with repressed genes. Comparison of the ATAC-seq signal at HNF-1β peaks showed that both activated and repressed genes associated with open chromatin. However, the enrichment of open chromatin was 30% higher at intragenic HNF-1β binding sites and 21% higher at distal intergenic sites in activated genes compared with repressed genes (Fig. 2c, Table S5). These differences were statistically significant (p = 2.3 × 10^–09^ and 1.5 × 10^–03^, respectively, Wilcoxon test). Similarly, the enrichment of H3K27ac was 37% higher at intragenic HNF-1β binding sites and 33% higher at distal intergenic sites in activated genes compared with repressed genes (p = 2.6 × 10^–19^ and 3.5 × 10^–04^, Wilcoxon test). The enrichment of H3K4me1 was 25% higher at intragenic sites and 15% higher at distal intergenic sites in activated genes compared with repressed genes (p = 3.9 × 10^–15^ and 2.3 × 10^–02^, Wilcoxon test). There were no significant differences between activated and repressed genes in ATAC-seq, H3K27ac, and H3K4me1 enrichment at HNF-1β binding sites in promotor regions (Fig. S2, Table S5). A total of 1577 HNF-1β genomic binding sites showed enrichment of both H3K27ac and H3K4me1. Of these sites, 738 were located in promoter regions, 531 were intragenic, and 308 were distal intergenic. Of the 1577 binding sites, 244 were located near genes that were activated by HNF-1β in the UB, while 160 were located near genes that were repressed by HNF-1β (Table S6).

The relationship between two repressive histone marks, H3K27me3 and H3K9me3, and HNF-1β binding was also examined. Only 136 H3K27me3 peaks overlapped with HNF-1β peaks, which was statistically significant (p < 2 × 10^–16^, hypergeometric test) but had a lower odds ratio (1.97) and much less overlap than ATAC-seq and activating histone modifications. Twenty H3K9me3 peaks overlapped with HNF-1β peaks, which was not statistically significant (p = 1, odds ratio = 0.54, hypergeometric test). There were no differences in H3K27me3 and H3K9me3 enrichment at HNF-1β binding sites in activated and repressed genes compared to genes with no change in expression (Fig. S5). Taken together, HNF-1β intragenic and distal intergenic peaks showed increased chromatin accessibility and activating histone marks in activated genes compared to repressed genes.

### HNF-1β peaks are associated with greater chromatin accessibility in UB-derived cell types in the developing kidney

**Figure 3:**
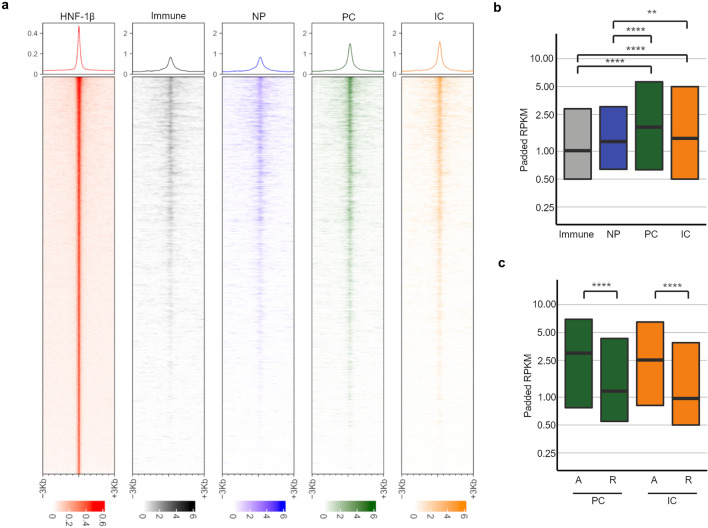
HNF-1β binding overlaps with regions of open chromatin in ureteric bud-derived cells. (**a**) Enrichment of single nuclear ATAC-seq (snATAC-seq) signal at sites of HNF-1β binding in chromatin from ureteric bud (UB)-derived principal cells (PC, green) and intercalated cells (IC, orange) in postnatal day (P)0 mouse kidneys. snATAC-seq in immune cells (gray) and nephron progenitors (NP, blue) is shown as a control. HNF-1β binding in UB cells is shown in red (left panel). Top panels show the total signal. (**b**) snATAC-seq enrichment at HNF-1β binding sites in immune cells, NP, PC, and IC shown in reads per kilobase of transcript (RPKM). Boxplots indicate the median and interquartile range. (**c**) snATAC-seq enrichment in PC and IC at HNF-1β binding sites near activated genes (A) and repressed genes (R). Asterisks indicate statistically significant differences using the Wilcoxon test (*p < 0.05; **p ≤ 0.01; ***p ≤ 0.001; ****p ≤ 0.0001). Median and mean enrichment values and individual Wilcoxon test results can be found in Supplementary Table S6.

Next, we determined whether the relationship between HNF-1β binding and chromatin accessibility seen in the embryonic kidney was observed in the UB. We took advantage of a single nuclear (sn)ATAC-seq analysis performed on postnatal day (P)0 mouse kidney^33^, an age at which kidney development is still ongoing. The enrichment of snATAC-seq signal from the UB-derived principal cells (PC) and intercalated cells (IC) was compared with immune cells and nephron progenitors (NP), which do not endogenously express HNF-1β. The enrichment of open chromatin at HNF-1β binding sites in PC was 47% higher than immune cells (p = 1.3 × 10^–54^, Wilcoxon test) and 41% higher than NP (p = 3.5 × 10^–13^, Wilcoxon test) (Fig. 3a). The enrichment of open chromatin at HNF-1β binding sites was 47% higher in IC than immune cells (p = 1.2 × 10^–29^, Wilcoxon test) and 41% higher than NP (p = 0.0023, Wilcoxon test) (Fig. 3b, Table S7).

We then compared the snATAC-seq signal at the HNF-1β binding sites in activated and repressed genes (Fig. 3c). The enrichment of open chromatin at HNF-1β binding sites in PC was 48% higher in activated genes than repressed genes (p = 8.5 × 10^–26^, Wilcoxon test). The enrichment of open chromatin at HNF-1β binding sites in IC was 43% higher in activated genes than repressed genes (p = 3.6 × 10^–24^, Wilcoxon test). Taken together, the association of ATAC-seq signal with HNF-1β binding sites seen in embryonic kidney was also observed in cells derived from the UB. Moreover, there was greater enrichment of open chromatin in genes that were activated by HNF-1β compared to repressed genes.

### HNF-1β regulates axon guidance genes in the developing kidney

**Figure 4:**
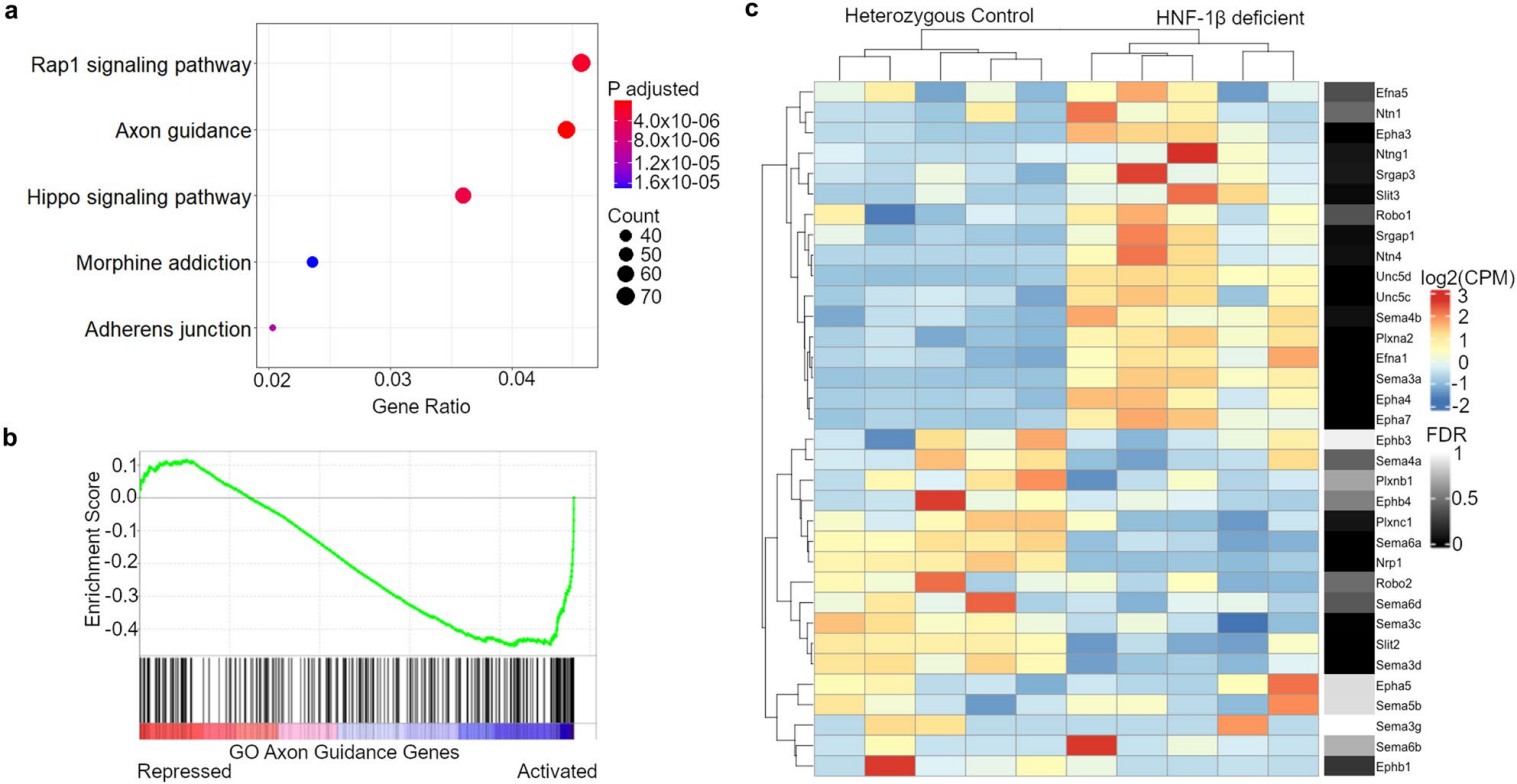
Axon guidance genes are regulated by HNF-1β in the developing ureteric bud. (**a**) Pathway analysis of HNF-1β gene targets identified by ChIP-seq in E14.5 mouse kidneys. (**b**) Gene set enrichment analysis (GSEA) showing that axon guidance is negatively enriched (Enrichment score = − 0.45, FDR adjusted p = 0.0) in HNF-1β-deficient ureteric bud (UB) cells compared to heterozygous control UB cells. (**c**) Heatmap showing expression (blue to red) of HNF-1β-regulated axon guidance genes in heterozygous control UB cells compared to versus HNF-1β-deficient UB cells, FDR p-values shown in gray.

To identify novel pathways that are regulated by HNF-1β in the developing mouse kidney, we performed KEGG^34–36^ analysis on the genes that were in closest proximity to the HNF-1β binding sites in E14.5 kidneys (Fig. 4a). Axon guidance (KEGG ID mmu04360) was identified as a significant canonical pathway (adjusted p-value = 7.13 × 10^–09^). The related Rap1 signaling pathway, which is involved in intracellular signaling for plexin axon guidance receptors^37^, was also identified. Axon guidance is a repulsive and attractant chemotaxis pathway that was first described in axon growth cones. More recently, the axon guidance signaling pathway has been identified in tubular epithelial cells where it has been implicated in branching morphogenesis in epithelial organs, including the kidney^38^. We found that HNF-1β binds near 68 axon guidance genes in the developing kidney. Of these genes, 34 encode ligands or receptors in the Semaphorin/Neuropilin/Plexin, Ephrin/Ephrin receptor, Slit/Robo, and Netrin/Unc families. Pathway analysis of RNA-seq data showed that axon guidance was also highly represented among the genes that were directly activated or repressed by HNF-1β (Fig. S6). Gene set enrichment analysis (GSEA) showed that the GO Axon Guidance pathway (GO:0007411) was enriched in the RNA-seq data from E14.5 UB cells (Enrichment score = − 0.45, FDR q-value = 0.0) (Fig. 4b). RNA-seq counts were ranked from the genes with the highest to lowest expression in HNF-1β mutant UB cells compared to heterozygous control UB cells. The enrichment score of − 0.45 indicated that the genes contributing most to the enrichment of the axon guidance pathway were genes that had lower expression in HNF-1β deficient cells, i.e. activated by HNF-1β. To confirm these findings, pathway analysis was performed on genes that were differentially expressed in HNF-1β-deficient mIMCD3 cells compared to wild-type mIMCD3 cells^39^. Mutant mIMCD3 cells contained the same deletion of *Hnf1b* exon 1 as HNF-1β mutant mice. Pathway analysis showed that axon guidance was the second most highly enriched pathway in HNF-1β mutant cells compared to wild-type cells (Table S8). These results indicate that HNF-1β regulates the transcription of axon guidance genes both in vitro in mIMCD3 cells and in vivo in developing kidney.

**Figure 5:**
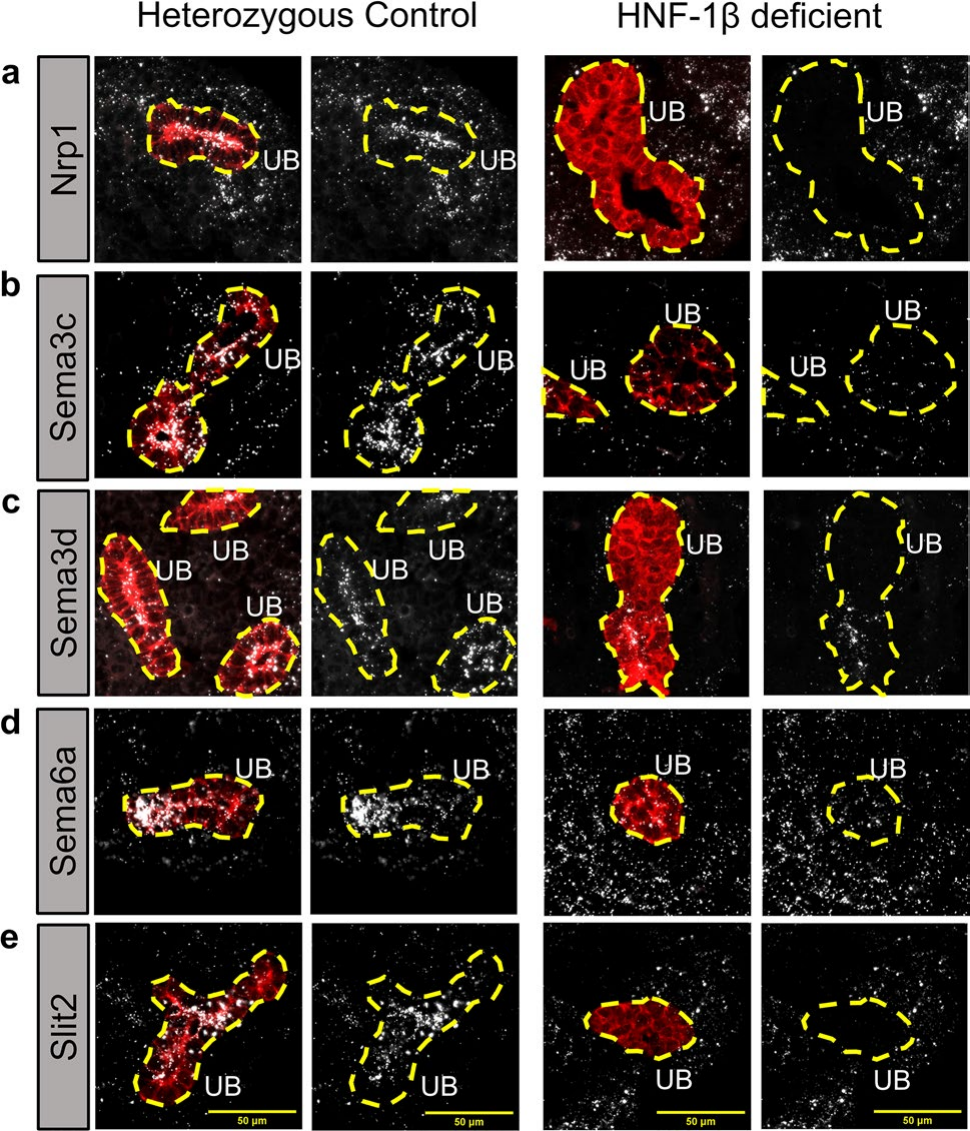
Dysregulation of axon guidance genes in HNF-1β mutant kidneys. Dual RNAscope-indirect immunofluorescence was performed on sagittal sections of E14.5 heterozygous control and HNF-1β mutant kidneys. Sections were stained using an anti-RFP (TdTomato) antibody (red) to visualize ureteric buds (UB). RNAscope in situ hybridization signals are shown in white. UBs are outlined in yellow dashed lines. RNAscope channels are shown alone for clarity on the right. Scale bar indicates 50 µm.

The changes in expression of HNF-1β gene targets in the ephrin, semaphorin, and Slit/Robo signaling pathways were investigated in greater detail. *Nrp1*, *Sema3c*, *Sema3d*, *Sema6a* and *Slit2* were significantly activated by HNF-1β, whereas *Efna1*, *Epha3*, *Epha4*, *Epha7*, *Ntn4*, *Plxna2*, *Sema3a*, *Sema4b*, *Slit3*, *Srgap1*, *Unc5c* and *Unc5d* were significantly repressed by HNF-1β (Fig. 4c). To validate RNA-seq results and investigate spatial expression of axon guidance genes bound by HNF-1β, fluorescent RNAscope in situ hybridization was performed on sagittal sections E14.5 heterozygous control and HNF-1β mutant kidneys. Kidney sections were co-stained with antibodies against RFP (TdTomato) to visualize the UB. *Nrp1* was expressed in the heterozygous control UB and MM. In the HNF-1β mutant kidney, expression of *Nrp1* decreased in the UB but remained unchanged in the MM, reflecting the UB-specific knockout of HNF-1β (Fig. 5a). *Sema3c*, *Sema3d*, and *Slit2* were highly expressed in the heterozygous control UB and weakly expressed in the surrounding MM. In the HNF-1β mutant kidney, expression was decreased in the UB but remained unchanged in the MM (Fig. 5b,c,e). *Sema6a* was expressed in heterozygous control UB and MM; expression decreased in the mutant UB and increased in the mutant MM (Fig. 5d).

Next, we examined the relationship between HNF-1β binding, ATAC-seq, and histone modifications at *Nrp1*, *Sema3c*, *Sema3d*, *Sema6a*, and *Slit2*. Nrp1, which was activated by HNF-1β (Fig. 4c, Table S4), contained an intragenic HNF-1β peak in intron 13 colocalized with H3K27ac, H3K4me1, and ATAC-seq enrichment (Fig. S7). *Sema6a*, which was activated by HNF-1β (Fig. 4c, Table S4), contained an intragenic HNF-1β binding site in its first intron that overlapped with H3K27ac and H3K4me1 peaks (Fig. S7). *Sema3c*, which was activated by HNF-1β (Fig. 4c, Table S4), did not contain an HNF-1β peak in the promoter, but there was an HNF-1β motif near the promoter and ATAC-seq, H3K4me3, and H3K27ac peaks were present near the promoter (Fig. S7). *Sema3d,* which was activated by HNF-1β (Fig. 4c, Table S4), did not contain an HNF-1β peak in the promoter, but ATAC-seq, H3K4me3, and H3K27ac peaks were present near the promoter (Fig. S7). *Slit2*, which was activated by HNF-1β (Fig. 4c, Table S4), contained 2 intragenic HNF-1β binding sites that did not overlap with ATAC-seq, H3K4me3, H3K27ac, or H3K4me1 peaks. *Slit2* did not have an HNF-1β binding site in the promoter, but ATAC-seq and H3K4me3 peaks were present near the promoter (Fig. S7). Taken together, HNF-1β regulates the expression of axon guidance genes in the UB and can either activate or repress gene transcription. Promoters of activated axon guidance genes also overlapped with ATAC-seq, H3K4me3 and H3K27ac peaks. Some intragenic HNF-1β peaks overlapped with both H3K27ac and H3K4me1 peaks.

## Discussion

The mechanisms whereby HNF-1β regulates branching morphogenesis during kidney development are incompletely understood. HNF-1β is required for Ret/GDNF signaling, a major pathway that regulates branching morphogenesis in the kidney. The MM expresses the ligand GDNF, which binds to the RET receptor and GFRA1 co-receptor that are expressed in the WD and UB^1,2^. HNF-1β regulates this pathway by directly activating transcription of *Gfra1* in the UB. To identify additional pathways that are regulated by HNF-1β, we conducted a genome-wide analysis of HNF-1β binding sites in the embryonic mouse kidney. We identified 8284 HNF-1β binding sites that were approximately equally distributed within promoters, gene bodies, and intergenic regions. Binding sites in the intragenic and intergenic regions likely corresponded to gene enhancers, a possibility that was supported by colocalization with epigenetic marks of active enhancers. To identify genes that were directly regulated by HNF-1β, we correlated HNF-1β binding measured by ChIP-seq with HNF-1β-dependent gene expression measured by RNA-seq in heterozygous control and HNF-1β mutant UB cells. This analysis identified 485 genes that were directly activated by HNF-1β and 526 genes that were directly repressed by HNF-1β.

We examined the global chromatin landscape of HNF-1β targets in the developing kidney by investigating chromatin accessibility and histone modifications at HNF-1β binding sites. We found that HNF-1β promoter peaks were highly enriched in open chromatin and activating marks H3K4me3 and H3K27ac (Fig. 2a). This finding indicated that HNF-1β promoter binding was associated with transcriptional activation^5,23,24,40^. We did not find a significant difference in HNF-1β binding or enrichment of open chromatin and activating histone marks at promoter peaks in differentially expressed HNF-1β targets (Fig. S2), which suggests that HNF-1β binding and histone modifications are required for both transcriptional activation and repression. This observation may be partly explained by the fact that regions of poised chromatin usually have high chromatin accessibility^41^. We found no significant overlap of HNF-1β peaks with repressive histone modifications H3K27me3 and H3K9me3 (Fig. S5), which suggests that H3K27me3 and H3K9me3 are not involved in HNF-1β-mediated transcriptional repression.

We found that there is greater ATAC-seq and H3K27ac enrichment in the intragenic and intergenic peaks of genes activated by HNF-1β. HNF-1β recruits CBP/P300 and PCAF to the targets it activates, which results in synergistic high levels of activation of its targets^7^. This study shows increased enrichment of H3K27ac in the genes HNF-1β activates compared to those it represses. This suggests that the mechanism in which HNF-1β activates its developmental targets is by histone acetylation. Additionally, H3K4me1 is also more highly enriched at intragenic and intergenic peaks of activated HNF-1β targets. This pattern is consistent with activated enhancers correlating with H3K27ac and H3K4me1 activity. H3K4me1 alone indicates primed enhancers, while dual H3K4me1 and H3K27ac indicates activated enhancers^31^. We found HNF-1β binding sites that have dual H3K27ac and H3K4me1 occupancy and are located near genes that are activated by HNF-1β in the UB (Table S6). This pattern suggests that HNF-1β also activates gene expression at enhancers in the intragenic and intergenic regions by facilitating the deposition of H3K27ac.

We also investigated the open chromatin landscape specifically in cells derived by the UB by comparing snATAC-seq data from immune cells and NPs versus ICs and PCs from P0 kidneys. We found that snATAC-seq enrichment at HNF-1β peaks in the UB-derived cell types is higher than in controls (Fig. 3a), suggesting that the open chromatin state is specifically associated with the presence of HNF-1β activity. Again, this pattern is consistent in for both genes activated and repressed by HNF-1β (Fig. 3b). Further, the snATAC-seq signal from IC and PC cells is significantly higher in activated genes versus repressed (Fig. 3c), suggesting that HNF-1β activation is correlated with increased chromatin accessibility. One limitation of our study is that we do not have ATAC-seq and histone modification data from HNF-1β-deficient UB cells. Future studies could investigate the dependence of chromatin accessibility and histone modifications on HNF-1β.

Pathway analysis showed that HNF-1β directly regulates axon guidance genes in the developing kidney. This result provides in vivo validation of previous in vitro studies in cultured mIMCD3 cells^42^. We measured the differential gene expression of the axon guidance targets of HNF-1β in heterozygous control and mutant UB cells by RNA-seq and RNAscope. Our results show that HNF-1β regulates axon guidance genes that have a known role in kidney development. We found that *Sema3c* expression is decreased in HNF-1β deficient E14.5 kidneys (Figs. 4c, 5c). *Sema3c* encodes the Sema3c secreted ligand, which interacts with receptors PlexinD1 and Nrp1^38,43^. Recombinant Sema3c can stimulate branching morphogenesis of mouse metanephros in ex-vivo organ culture, and conversely, Sema3a/Sema3c double null metanephros show decreased branching morphogenesis compared to wild-type^44^. HNF-1β upregulates the expression of *Sema3c* in the developing kidney to stimulate branching morphogenesis. *Nrp1* is downregulated in HNF-1β deficient kidneys. Previous studies have shown that Nrp1 stimulates branching morphogenesis in mIMCD3 cells and mouse proximal tubule cells^45^. This suggests that HNF-1β upregulates *Nrp1* expression to increase branching morphogenesis. The putative ligand-receptor pairs of the axon guidance genes regulated by HNF-1β are expressed in neighboring cell populations in the developing kidney, and some are also expressed in the UB^46^. This finding suggests that HNF-1β may regulate both autocrine and paracrine axon guidance signaling to control branching morphogenesis, a possibility that may be explored in future studies.

We examined the ATAC-seq, H3K4me3, H3K27ac, and H3K4me1 enrichment at HNF-1β binding sites near axon guidance genes. Although we were not able to find a global pattern of histone modification enrichment, some genes have histone modification patterns consistent with promoter or enhancer activation. We found that the *Nrp1* intragenic peak and *Sema6a* intragenic peak overlap with H3K27ac and H3K4me1 signals, which suggests that HNF-1β may activate these genes by binding to enhancer regions. We also observed ATAC-seq, H3K4me3, and H3K27ac signal near the promoters of *Sema3c*,* Sema3d*, and* Slit2*, even though these genes do not have an HNF-1β peak near the promoter. All of these genes also are expressed outside of the UB in both control and HNF-1β deficient kidneys (Fig. 5b–e). These promoter histone modifications in the absence of an HNF-1β binding site could be due to their activation by another transcription factor.

In summary, our study identifies novel HNF-1β genomic binding sites in the developing kidney as well as global patterns of HNF-1β-dependent transcriptional regulation associated with histone modifications. We identified axon guidance genes that are directly regulated by HNF-1β in the developing kidney. Our findings suggest that the branching morphogenesis defects seen in HNF-1β deficient kidneys may be attributed to the dysregulation of axon guidance genes.

## Methods

### Transgenic mice

Hoxb7/Cre mice^47^ expressing Cre recombinase under the control of the UB-specific Hoxb7 promoter were obtained from the Jackson Laboratory (Stock #004692). *Hnf1b*^fl/+^ mice^48^ containing loxP sites flanking exon 1 of the HNF-1β gene were a generous gift from Dr. Marco Pontoglio (Cochin Institute). Gt(Rosa)26Sor^tm9(CAG-tdTomato)Hze^ mice^49^ (tdT mice) containing a tdTomato reporter gene that is activated by Cre/loxP recombination were obtained from the Jackson Laboratory (Stock #007909). The genetic backgrounds of the mice were CFW (Hoxb7/Cre) and C57BL/6 (*Hnf1b*^fl/+^ and tdT).

Hoxb7/Cre;*Hnf1b*^fl/+^ mice were crossed with *Hnf1b*^fl/fl^;tdT mice to generate Hoxb7/Cre;*Hnf1b*^fl/fl^;tdT embryos in which HNF-1β was specifically inactivated in the UB. Hoxb7/Cre;*Hnf1b*^fl/+^;tdT littermates do not have a kidney phenotype and were used as controls. The morning of detection of the copulation plug was considered embryonic day (E)0.5. On E14.5, pregnant dams were euthanized via carbon dioxide according to the American Veterinary Medical Associat guidelines, and embryos were removed and placed in phosphate buffered saline (PBS) on ice. Embryonic kidneys were dissected using a Leica M420 microscope. Embryos of both sexes were used. All experiments involving animals were approved by the Institutional Animal Care and Use Committee at the University of Minnesota and complied with ARRIVE guidelines^50^. All methods were performed in accordance with the relevant guidelines and regulations.

### ChIP-sequencing

Kidneys were dissected from wild-type C57BL/6J embryos at E14.5, pooled, and cross-linked in 2% paraformaldehyde (PFA) in PBS for 10 min at room temperature followed by neutralization in 0.125 M glycine for 5 min at room temperature. Cross-linked kidneys were transferred into lysis buffer (0.05 M HEPES–NaOH (pH 7.5), 0.14 M NaCl, 1 mM EDTA, 10% glycerol, 0.5% NP-40, 0.25% Triton X-100), and nuclei were isolated using a 1 ml tight Dounce homogenizer. Chromatin shearing and immunoprecipitation (IP) were performed using buffers from the Millipore EZChIP kit (Sigma-Aldrich). Nuclei were pelleted at 2300×*g* for 5 min and were transferred into SDS lysis buffer (1% SDS, 10 mM EDTA, 50 mM Tris–HCl (pH 8.0)). Samples were incubated on ice for 10 min, and the chromatin was sheared using a Covaris S220 focused-ultrasonicator for 7.5 min at 7.5 Watts, peak power 75, and duty factor 10. The chromatin was cleared by centrifugation at 15,000×*g* at 4 °C for 10 min, and the chromatin was diluted 1:10 into ChIP dilution buffer (0.01% SDS, 1.1% Triton X-100, 1.2 mM EDTA, 16.7 mM Tris–HCl (pH 8.1), 167 mM NaCl). A 1% input sample was removed, and HNF-1β was immunoprecipitated using 5 μg rabbit anti-HNF-1β antibody at 4 °C overnight with rotation.

Protein A/G beads (40 μl, Santa Cruz) were added to the immunoprecipitates and incubated at 4 °C for 3 h with rotation. Beads were pelleted by centrifugation at 4000×*g* for 1 min then were rinsed sequentially with a low salt immune complex wash buffer (0.1% SDS, 1% Triton X-100, 2 mM EDTA, 20 mM Tris–HCl (pH 8.0), 150 mM NaCl), high salt immune complex wash buffer (0.1% SDS, 1% Triton X-100, 2 mM EDTA, 20 mM Tris–HCl (pH 8.0), 500 mM NaCl), LiCl immune complex wash buffer (0.25 M LiCl, 1% NP-40, 1 mM EDTA, 10 mM Tris–HCl (pH 8.0)), and twice with TE buffer (10 mM Tris–HCl (pH 8.0), 1 mM EDTA). Each wash was performed at 4 °C with rotation for 5 min. Protein-chromatin complexes were eluted twice by incubation in 100 μl elution buffer (1% SDS, 0.1 M NaHCO_3_) at 37 °C for 15 min with rotation. Beads were pelleted at 4000×*g* for 1 min at room temperature, and the eluate was collected. Cross-linking was reversed by overnight treatment with 8 μl 5 M NaCl at 55 °C, then samples were incubated with 4 μl 0.5 M EDTA, 1 μl RNase A (10 mg/ml), and 1 μl Proteinase K (10 mg/ml) for 2 h at 65 °C. Chromatin was purified using Qiagen MinElute PCR Purification Kits.

Sequencing libraries were prepared using the KAPA Hyper Prep Kit (Roche). Chromatin was subjected to an end-repair poly-A tail reaction and ligated to the xGen Stubby Adaptor (Integrated DNA Technologies) at 1:50 dilution. Ligated samples were purified with 0.8 × volume of KAPA Pure beads (Roche). To determine the number of amplification samples, a 5% test library amplification was carried out by quantitative PCR (qPCR) with the Illumina universal P2 primer and one Illumina index primer for 40 cycles. Three cycles were added to the threshold cycle number (C_t_) for the final library amplification (16 cycles for input samples, 20 cycles for IgG and anti-HNF-1β immunoprecipitates). Libraries were quantitated using Qubit and were selected for 200–400 base pair fragments using Invitrogen 2% agarose E-Gels. Indexed libraries were sequenced on an Illumina Next-seq 1 × 75-bp high-output run.

### RNA-sequencing

Kidneys were dissected from heterozygous control and HNF-1β mutant embryos collected from several pregnant dams at E14.5 and dissociated into single-cell suspensions by incubating in 125 U/ml type 1 collagenase (Sigma-Aldrich) in PBS at 37 °C for 15 min with mixing every 5 min. Samples were centrifuged at 845×*g* and incubated in 0.25% Trypsin–EDTA (Gibco) at 37 °C for 15 min with mixing every 5 min. After neutralizing with 20% fetal bovine serum and washing in PBS, single-cell suspensions were incubated with near-infrared live/dead dye (ThermoFisher) at 1:1000 dilution for 5 min. TdTomato-positive live cells were purified using a BD FACSAria cell sorter. Cells were sorted directly into Qiazol RNA lysis buffer. Total RNA was extracted with the Qiagen miRNeasy kit using NEB Monarch RNA Cleanup Columns to elute into a low volume of 8 µl in H_2_O.

cDNA libraries were prepared using the NEBNext Single Cell/Low Input RNA Library Prep Kit for Illumina. RNA concentration and RNA integrity (RIN) scores were determined using an Agilent 4200 Tapestation. All sample RIN scores were ≥ 8.0. Libraries were prepared in the same batch, and the nanogram amount of the least concentrated RNA sample was used in each reaction. Samples were reverse transcribed with a template switching reverse transcriptase, and cDNA was amplified for 12 PCR cycles. Samples were purified using Ampure XP SPRI beads (Beckman). cDNA samples were fragmented using the NEBNext Ultra II FS Enzyme Mix. The NEBNext Adaptor for Illumina was ligated onto fragmented cDNA at a 1:25 dilution. Samples were purified using SPRI beads, and libraries were amplified with Illumina sequencing primers. Samples were purified using SPRI beads and eluted into 0.1 × TE buffer. Libraries were sequenced on an Illumina NextSeq 2 × 75-bp mid-output run.

### RNAscope in situ hybridization

Kidneys were dissected from heterozygous control and HNF-1β mutant embryos at E14.5 and fixed in 4% PFA in PBS for 15 min at room temperature. Kidneys were transferred to 30% sucrose in PBS solution, embedded in Optimal Cutting Temperature compound, frozen and stored at − 80 °C. 4 μm-thick sagittal cryosections were prepared and mounted on glass slides. RNAscope in situ hybridization was performed using the RNAscope Multiplex Fluorescent V2 Assay kit (ACD). Slides were washed in PBS for 5 min and baked at 60 °C for 20 min. Slides were incubated in 4% PFA in PBS for 15 min at 4 °C then dehydrated in 50% ethanol for 5 min, 70% ethanol for 5 min, and 100% ethanol for 5 min twice at room temperature. Slides were air-dried and incubated with 10% H_2_O_2_ for 10 min at room temperature. After rinsing in distilled water three times, slides were incubated in ACD heat retrieval buffer for 5 min at 95 °C. Slides were rinsed in distilled water three times and incubated in 100% ethanol for 3 min at room temperature. Slides were air dried and incubated with ACD protease for 30 min at 40 °C in a humidified slide moat. Samples were incubated with RNAscope probes for 2 h at 40 °C. Slides were washed twice in ACD wash buffer for 2 min at room temperature after probe incubation and between each amplification step. Samples were incubated sequentially with Amp1 for 30 min at 40 °C, Amp2 for 30 min at 40 °C, Amp3 for 15 min at 40 °C, HRP-C1 for 15 min at 40 °C, the Opal570 reagent (Akoya) diluted 1:1000 in TSA buffer (ACD) for 30 min at 40 °C, and HRP blocker for 15 min at 40 °C.

### Indirect immunofluorescence

Kidney sections were blocked in Rodent Block M (Biocare Medical) for 1 h at room temperature. Samples were incubated with a rabbit anti-RFP (TdTomato) primary antibody (1:1000) overnight at 4 °C followed by a secondary antibody (1:1000) for 2 h at room temperature. DAPI stain solution (ACD) was applied, and samples were mounted with Prolong Gold Antifade Mountant (ThermoFisher). Photomicrographs were acquired with a Leica DM5500 B upright microscope equipped with a DFC7000 T camera. Brightness and contrast were adjusted equally across all images, including controls, using FIJI/ImageJ.

### Antibodies and reagents

The primary antibodies used in this study were rabbit anti-HNF-1β (Santa Cruz, Cat #22840) and rabbit anti-RFP (TdTomato) (Rockland, Cat #600-401-379). Alexa Fluor 647-conjugated donkey anti-rabbit IgG (Jackson Immunoresearch, Cat #711-506-152) was used as a secondary antibody.

RNAscope was performed using the following probes: Efna1 (ACD, Cat #428621), Nrp1 (ACD, Cat #471621), Sema3c (ACD, Cat #441441), Sema3d (ACD, Cat #488111), Sema6a (ACD, Cat #508101), and Slit2 (ACD, Cat #449691).

### Data analysis

ChIP-sequencing reads were trimmed using Trimmomatic (v 0.32)^51^ using the TruSeq3-SE.fa:2:30:10 leading:3 trailing:3 slidingwindow:4:5 minlen:25 parameters. ChIP-seq read mapping was performed via BWA MEM (0.7.17)^52^ using the mouse genome (GRCm38) as a reference. ChIP peaks were identified using MACS (2.1.1.20160309)^53^. Peak lists from two independent experiments were merged using R (4.1.2)^54^ to find high confidence peaks present in both datasets. ChIP-sequencing quality analysis was performed using Phantompeakqualtools^55,56^ and is included in Supplementary Table S1. Peaks were assigned to the nearest gene promoter. For peaks close to small pseudogenes within the gene body of a known target, the peak was reassigned to the known target gene. The Integrative Genome Viewer (IGV)^57^ was used to visualize ChIP-seq peaks. Meme Suite 5.0.1^58^ was used to perform de novo motif analysis. ClusterProfiler^59^ was used to perform KEGG pathway analysis, and EnrichedHeatmap^60^ was used to make heatmaps. ggplot2^61^ was used to create figures.

RNA-sequencing reads were trimmed using Trimmomatic (v 0.33) enabled with the optional “-q” option; 3 bp sliding-window trimming from 3′ end requiring minimum Q30. Quality control on raw sequence data for each sample was performed with FastQC^62,63^. Read mapping was performed via Hisat2 (v2.1.0)^64^ using the mouse genome (GRCm38) as a reference. Gene quantification was performed using Feature Counts for raw read counts. Differentially expressed genes were identified using the edgeR^65–67^ (negative binomial) feature in CLCGWB (Qiagen, Redwood City, CA) with raw read counts. ClusterProfiler was used for KEGG pathway analysis, and the GSEA software^68,69^ was used to perform gene set enrichment analysis. Ingenuity Pathway Analysis was performed on differentially-expressed genes (GSE97770) with log_2_ fold-change > 1 or < -1 in HNF-1β-deficient mIMCD3 cells compared to wild-type mIMCD3 cells^39^.

H3K4me3, H3K27ac, H3K4me1, H3K27me3, and H3K9me3 ChIP-seq data and ATAC-seq data was obtained from ENCODE^27,28^. The overlap of each histone mark and open chromatin with HNF-1β binding sites was tested using hypergeometric tests. Enrichment of each mark at HNF-1β binding sites was examined by counting the number of reads within 1 kb of each HNF-1β peak. Read counts were normalized to peak width and read depth to generate reads per kilobase of transcript (RPKM) values for each HNF-1β peak.

### Statistical analysis

The Mann–Whitney Wilcoxon nonparametric test was used for pairwise comparisons. p < 0.05 was considered significant. The hypergeometric test was used to test overlap between ChIP-seq datasets, where p < 0.05 was considered significant.

## Supplementary Information

Supplementary Information 1.

Supplementary Information 2.

Supplementary Information 3.

## Data Availability

E14.5 ChIP-sequencing and E14.5 UB RNA-sequencing data have been deposited into NCBI’s Gene Expression Omnibus (GEO)^70^ with accession numbers GSE205189 and GSE205217. E14.5 H3K4me3 ChIP-seq (ENCSR669AQL), E14.5 ATAC-seq (ENCSR758IRM)**,** E14.5 H3K27ac ChIP-seq (ENCSR057SHA), E14.5 H3K4me1 ChIP-seq (ENCSR196ENU), E14.5 H3K27me3 ChIP-seq (ENCSR399UVI), and E14.5 H3K9me3 ChIP-seq (ENCSR405TGI) datasets were obtained from ENCODE^27,28^.
