# Supplementary material for: Multiomics analysis reveals that hepatocyte nuclear factor 1β regulates axon guidance genes in the developing mouse kidney
Source: Sci Rep. 2022 Oct 20;12:17586. doi: 10.1038/s41598-022-22327-5 (PMC9585060; doi:10.1038/s41598-022-22327-5)
Supplement: Supplementary file 1 — Supplementary Information 1. [file 41598_2022_22327_MOESM1_ESM.docx]

**Supplement to: Multiomics analysis reveals that hepatocyte nuclear factor 1β regulates axon guidance genes in the developing mouse kidney**

Annie Shao^1,2^, Micah D Gearhart^3^, Siu Chiu Chan^1^, Zhen Miao^4^, Katalin Susztak^4^, and *Peter Igarashi^1,2^

*Address for correspondence: Peter Igarashi, M.D., Department of Medicine, University of Minnesota Medical School, 420 Delaware Street SE, MMC 194, Minneapolis, MN 55455, USA, igarashi@umn.edu

^1^Department of Medicine, University of Minnesota Medical School, Minneapolis, Minnesota, USA

^2^Molecular, Cellular, Developmental Biology and Genetics Graduate Program, University of Minnesota, Minneapolis, Minnesota, USA

^3^Department of Genetics, Cell Biology and Development, University of Minnesota, Minneapolis, Minnesota, USA

^4^Renal, Electrolyte, and Hypertension Division, Department of Medicine, University of Pennsylvania, Perelman School of Medicine, Philadelphia, Pennsylvania, USA


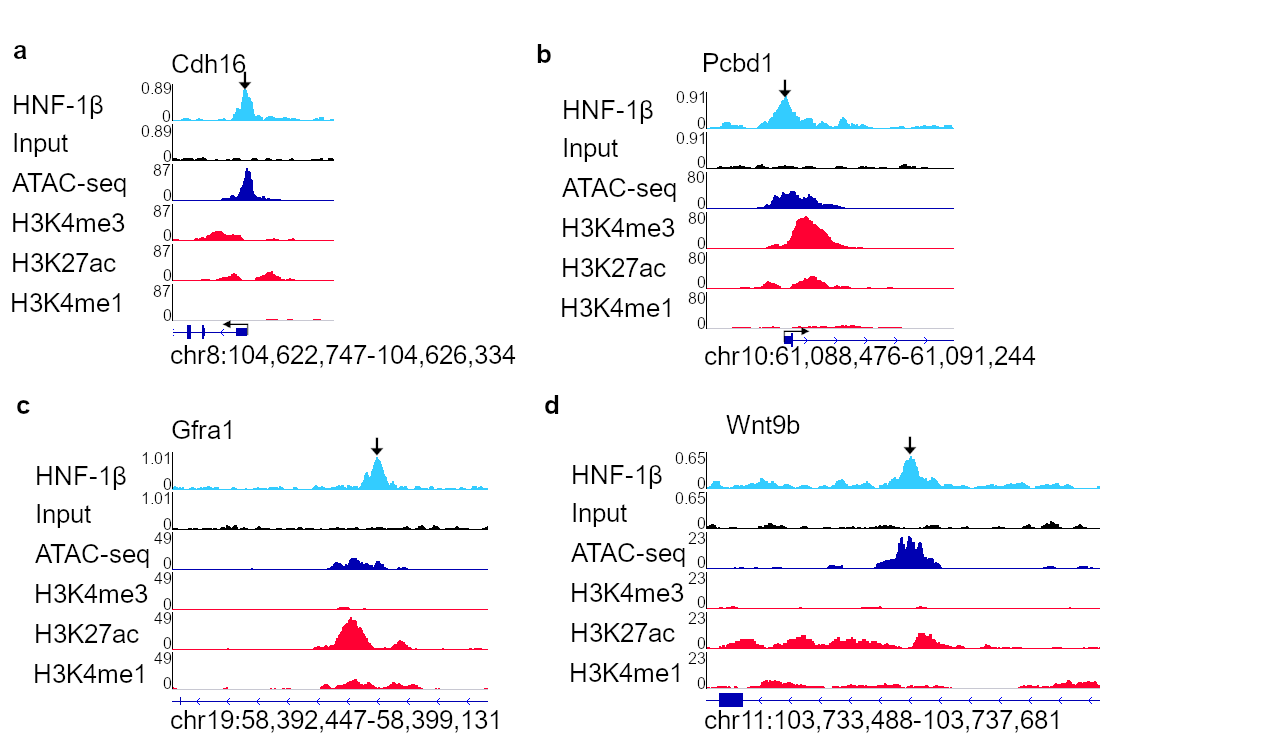


**Supplementary Figure 1. Open chromatin and activating histone marks at known HNF-1β binding sites.** HNF-1β binding, input DNA, ATAC-seq, H3K4 trimethylation (H3K4me3), H3K27 acetylation (H3K27ac), and H3K4 monomethylation (H3K4me1) are shown for the *Cdh16* promoter (A), *Pcbd1* promoter (B), *Gfra1* intron 4 (C), and *Wnt9b* intron 1 (D). Thin blue lines indicate introns, and thick blue lines indicate exons. The transcription start site and direction of transcription are indicated by the bent arrow. HNF-1β ChIP-seq peaks are indicated with vertical arrows.

**
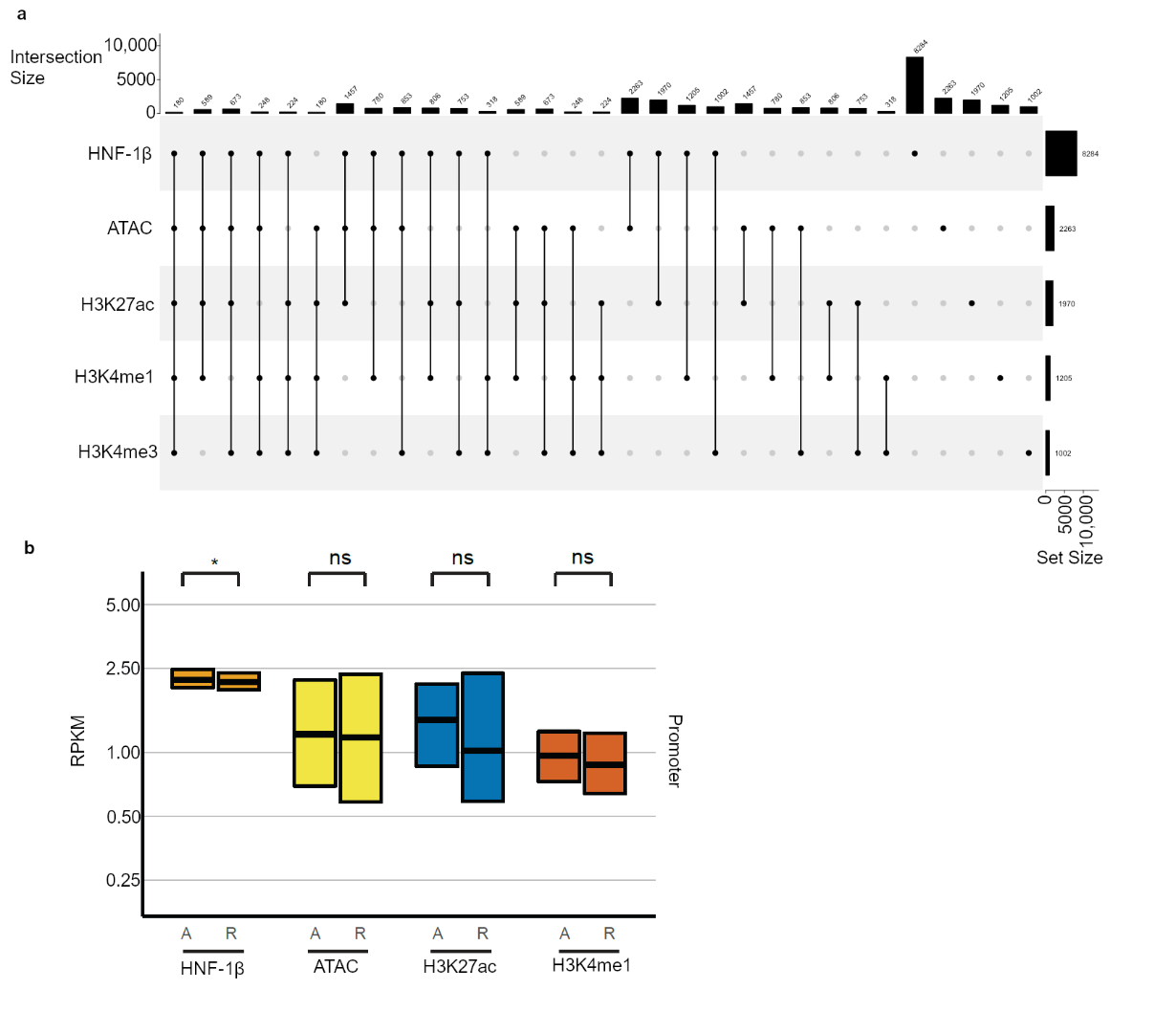
**

**Supplementary Figure 2. Comparison of epigenetic marker enrichment at HNF-1β binding sites.** A) Overlap of ATAC-seq, H3K27 acetylation (H3K27ac), H3K4 monomethylation (H3K4me1), and H3K4 trimethylation (H3K4me3) with HNF-1β binding peaks. The total number of peaks in each category is shown in the histograms. B) Comparison of HNF-1β binding, ATAC-seq signal, H3K27ac, and H3K4me1 in genes that are directly activated (A) or repressed (R) by HNF-1β. Binding in promoter regions is shown. Normalized enrichment values are shown as reads per kilobase of transcript (RPKM). Boxplots indicate the median and interquartile range. Asterisks indicate statistically significant differences using the Wilcoxon test (*, p<0.05. ns, p≥0.05). Median and mean enrichment values and individual Wilcoxon test results can be found in Supplementary Table 5.


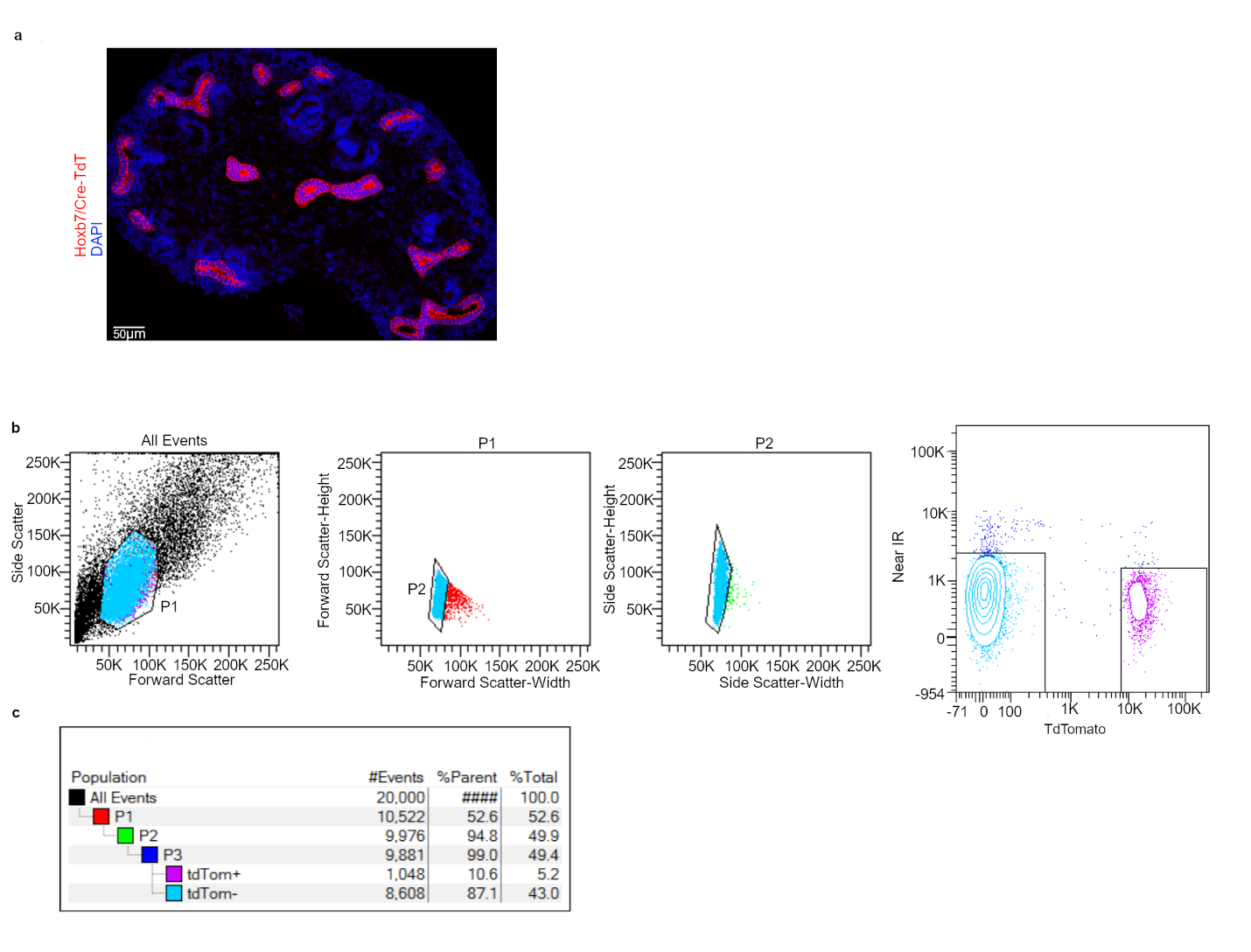


**Supplementary Figure 3. Isolation of ureteric bud cells from heterozygous control E14.5 mouse kidneys.** A) Sagittal section of a Hoxb7/Cre;*Hnf1b*^fl/+^;tdT E14.5 kidney stained with anti-RFP (TdTomato) antibody (red). DAPI nuclear staining is shown in blue. Scale bar indicates 50 µm. B) Flow cytometry forward and side scatter used to purify single cells. Right panel shows gating of TdTomato fluorescence to purify UB cells (red) from non-UB controls (blue). C) Yield of cells is shown as numbers and percentages.


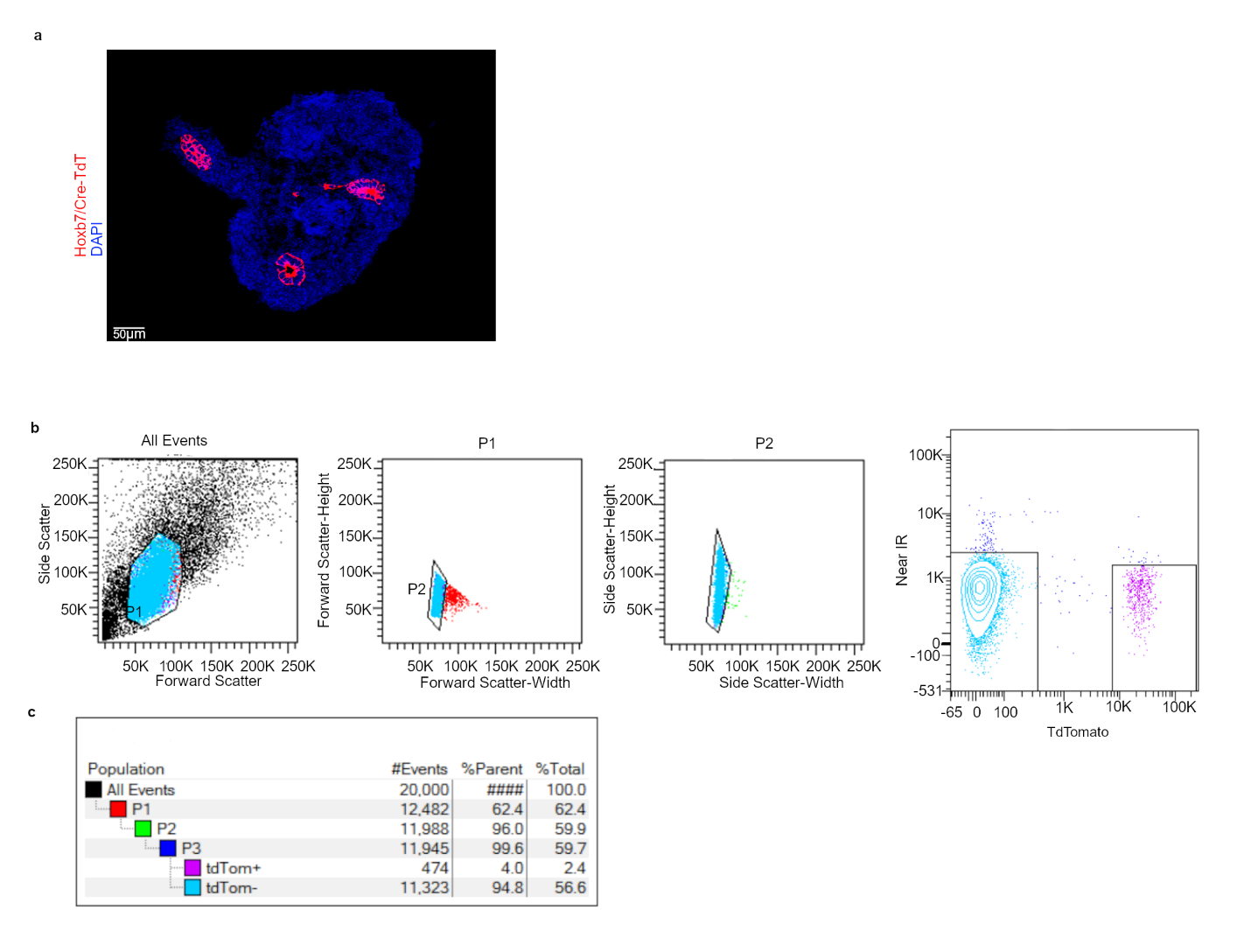


**Supplementary Figure 4. Isolation of UB cells from HNF-1β mutant E14.5 mouse kidneys.** A) Sagittal section of a Hoxb7/Cre;*Hnf1b*^fl/fl^;tdT E14.5 kidney stained with anti-RFP (TdTomato) antibody (red). DAPI nuclear staining is shown in blue. Scale bar indicates 50 µm. B) Flow cytometry forward and side scatter used to purify single cells. Right panel shows gating of TdTomato fluorescence to purify UB cells (red) from non-UB controls (blue). C) Yield of cells is shown as numbers and percentages.


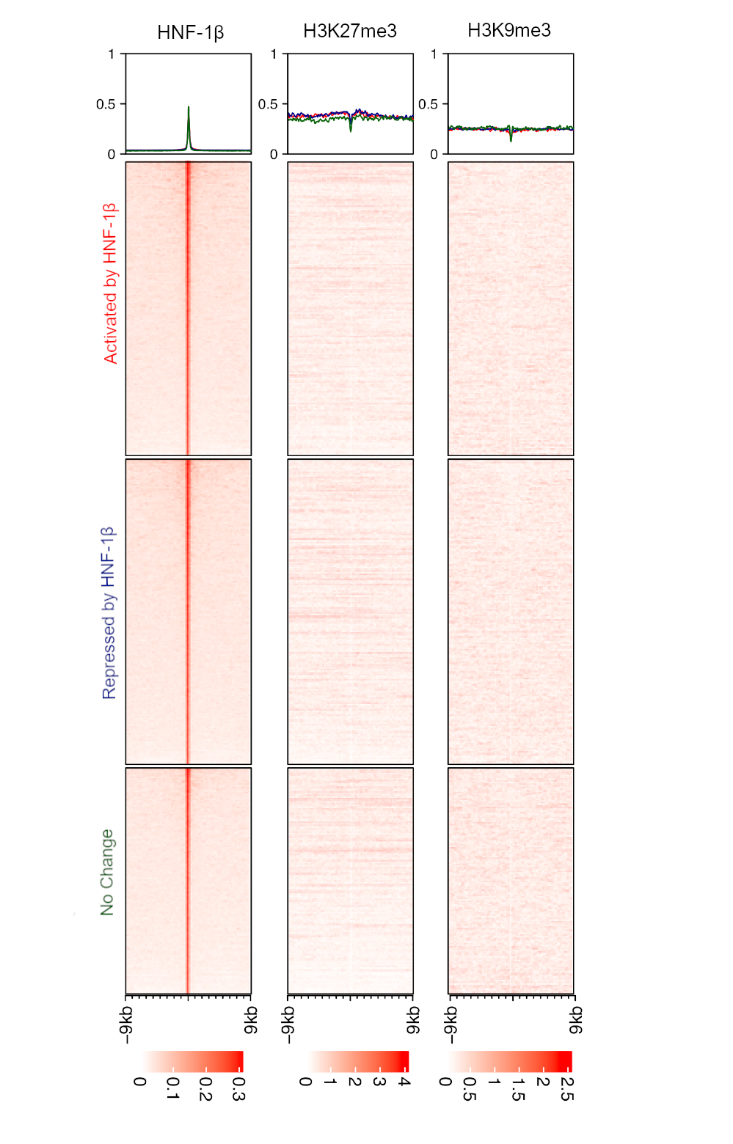


**Supplementary Figure 5. Lack of enrichment of repressive histone marks at HNF-1β binding sites.** Enrichment of H3K27 trimethylation (H3K27me3) and H3K9 trimethylation (H3K9me3) at HNF-1β binding sites in chromatin from E14.5 kidney. Enrichment is shown for genes that are activated by HNF-1β (upper panel), repressed by HNF-1β (middle panel), or unchanged in expression (lower panel). Peaks are centered on HNF-1β binding sites (left panels). Top panel shows total binding in the activated (red line), repressed (blue line), and unchanged genes (green line).

**
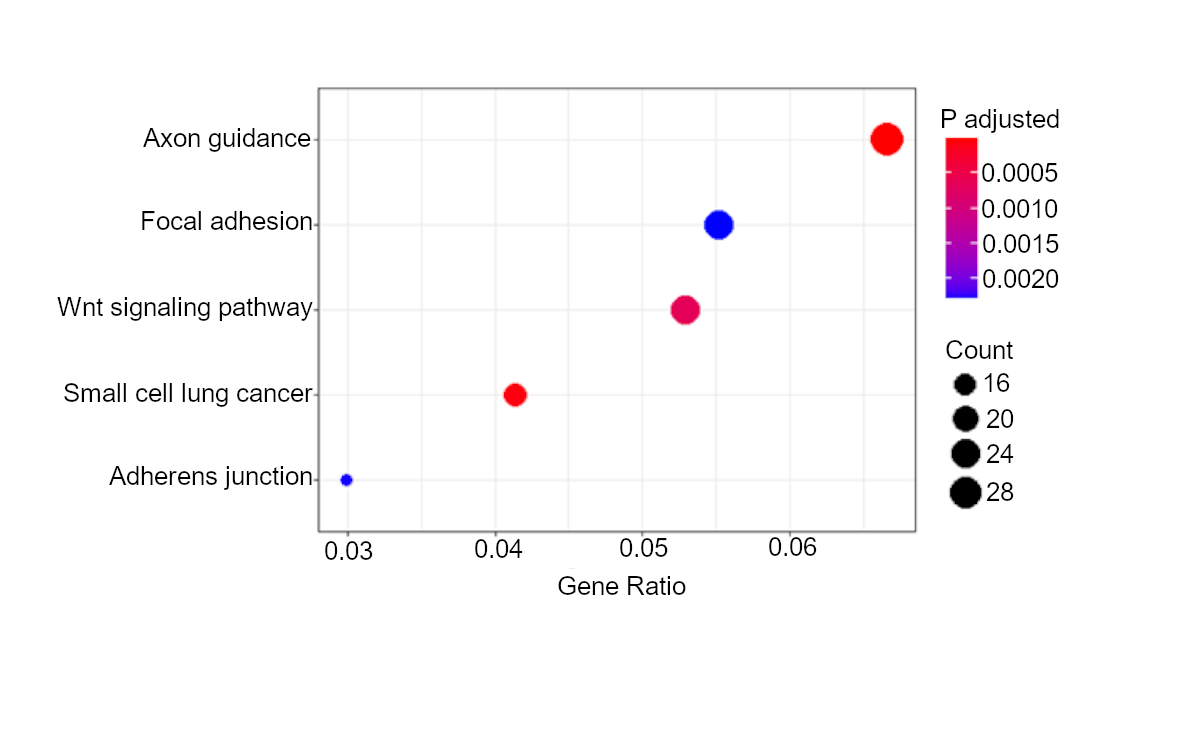
**

**Supplementary Figure 6. Pathway analysis of HNF-1β gene targets in E14.5 kidney.** Pathway analysis was performed on genes that contained nearby HNF-1β binding sites by ChIP-seq and were also differentially expressed in HNF-1β mutant UB cells compared to heterozygous control UB cells.


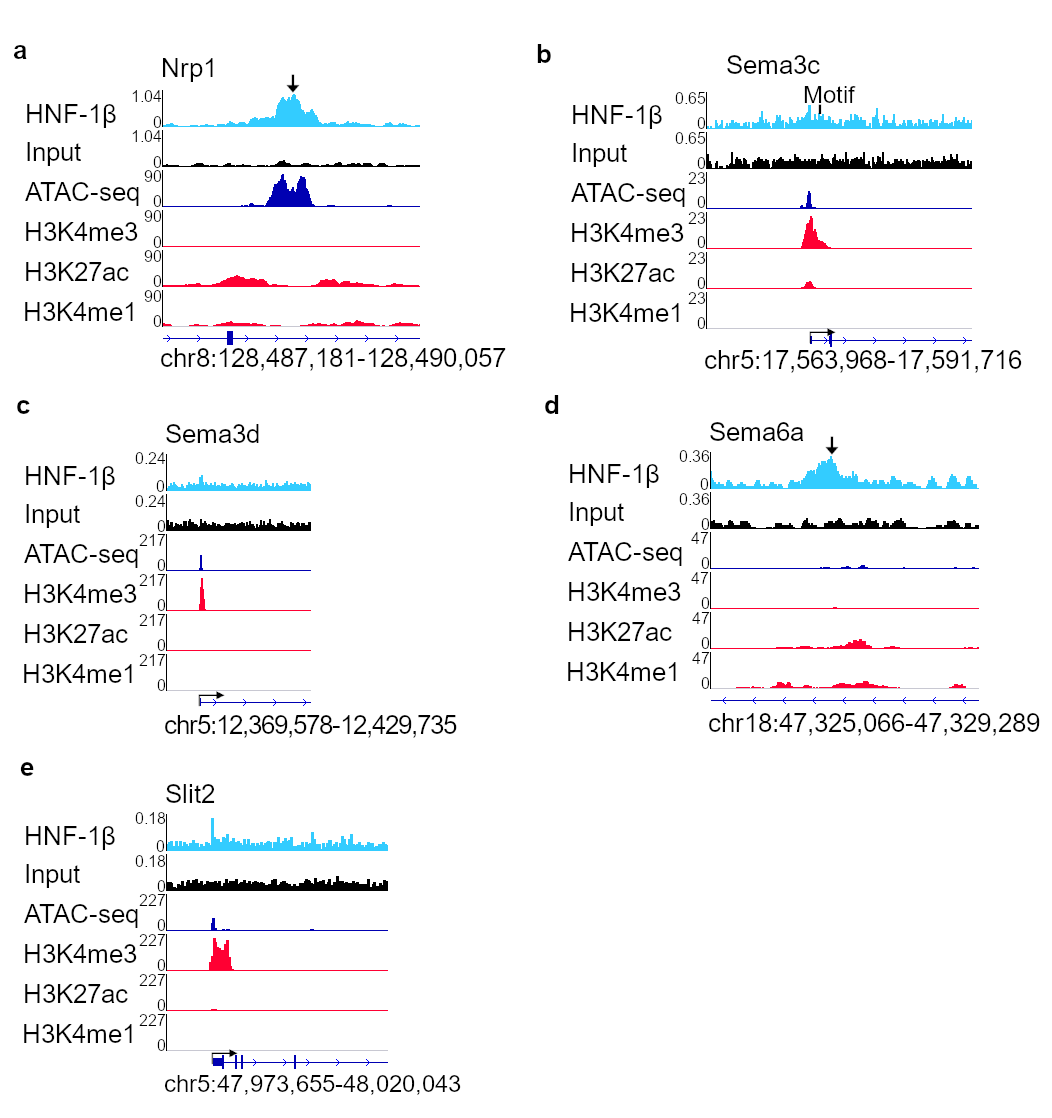


**Supplementary Figure 7. Open chromatin and activating histone marks at HNF-1β binding sites in axon guidance genes.** HNF-1β binding, input DNA, ATAC-seq, H3K4 trimethylation (H3K4me3), H3K27 acetylation (H3K27ac), and H3K4 monomethylation (H3K4me1) are shown for *Nrp1* intron 13 (A), *Sema3c* promoter (B), *Sema3d* promoter (C), *Sema6a* intron 1 (D), and *Slit2* promoter (E). Thin blue lines indicate introns, and thick blue lines indicate exons. The transcription start site and direction of transcription are indicated by the bent arrow. HNF-1β ChIP-seq peaks are indicated with vertical arrows.

| Sample | Starting Reads | Uniquely Mapped Reads | Nonredundant Fraction (NRF) | NSC | RSC | Fraction of Reads in Peaks (FRiP) |
| --- | --- | --- | --- | --- | --- | --- |
| HNF-1β replicate 1 | 81030889 | 20132359 | 0.29 | 1.03 | 1.26 | 2.50 |
| Input replicate 1 | 34536260 | 24472358 | 0.71 | 1.01 | 0.89 |  |
| HNF-1β replicate 2 | 90201392 | 21256370 | 0.29 | 1.03 | 1.35 | 1.35 |
| Input replicate 2 | 36540752 | 25711586 | 0.71 | 1.01 | 1.02 |  |

**Supplementary Table 1. ChIP-seq quality analysis.** Starting reads, uniquely mapped reads, nonredundant fraction (NRF), normalized strand cross-correlation (NSC), relative strand cross-correlation (RSC), and fraction of reads in peaks for two biological replicates of HNF-1β ChIP-seq in E14.5 kidneys.

| Gene Classification | Promoter | Intragenic | Distal Intergenic | Total |
| --- | --- | --- | --- | --- |
| Protein Coding | 1509 | 2592 | 967 | 5068 |
| Processed Pseudogene | 162 | 5 | 902 | 1069 |
| lncRNA | 166 | 270 | 546 | 982 |
| Antisense | 72 | 88 | 52 | 212 |
| Unannotated | 60 | 3 | 119 | 182 |
| snRNA | 39 | 0 | 102 | 141 |
| miRNA | 39 | 0 | 95 | 134 |
| snoRNA | 22 | 0 | 106 | 128 |
| Processed Transcript | 35 | 41 | 46 | 122 |
| Unprocessed Pseudogene | 14 | 4 | 48 | 66 |
| Miscellaneous RNA | 7 | 0 | 39 | 46 |
| Transcribed Processed Pseudogene | 8 | 6 | 16 | 30 |
| rRNA | 3 | 0 | 19 | 22 |
| Bidirectional Promoter lncRNA | 2 | 1 | 8 | 11 |
| Transcribed Unprocessed Pseudogene | 1 | 5 | 4 | 10 |
| Polymorphic Pseudogene | 2 | 5 | 2 | 9 |
| Unitary Pseudogene | 2 | 0 | 7 | 9 |
| Sense Intronic | 7 | 1 | 0 | 8 |
| Sense Overlapping | 0 | 7 | 0 | 7 |
| Pseudogene | 2 | 0 | 4 | 6 |
| scaRNA | 0 | 0 | 5 | 5 |
| Transcribed Unitary Pseudogene | 1 | 3 | 1 | 5 |
| IG V gene | 2 | 0 | 1 | 3 |
| TR V gene | 1 | 0 | 2 | 3 |
| Ribozyme | 0 | 0 | 2 | 2 |
| TR V Pseudogene | 0 | 0 | 2 | 2 |
| sRNA | 0 | 0 | 1 | 1 |
| TR C gene | 0 | 0 | 1 | 1 |

**Supplementary Table 2. Gene classification of HNF-1β targets in E14.5 kidneys.** Number of HNF-1β peaks in promoter, intragenic, and distal intergenic regions and total number of peaks are shown for each gene class. Genes were assigned based on nearest proximity to HNF-1β binding sites. Gene classifications were defined by Ensembl.

| Sample Name | Raw Count | Trimmed Count |
| --- | --- | --- |
| Mutant m7641-2 | 19102076 | 18451895 |
| Mutant m7641-3 | 19617748 | 19003173 |
| Mutant m8867-8 | 21765630 | 21009362 |
| Mutant m9072-5 | 22139248 | 21483206 |
| Mutant m9072-7 | 21311410 | 20576424 |
| Wild-type w7641-1 | 18884817 | 18195948 |
| Wild-type w7641-5 | 16475092 | 15893519 |
| Wild-type w8867-5 | 18436501 | 17720074 |
| Wild-type w9072-1 | 22035885 | 21348846 |
| Wild-type w9072-2 | 22488116 | 21798231 |

**Supplementary Table 3. Read depth of RNA-seq samples.** Raw read count and trimmed read count of RNA-seq data from heterozygous control and HNF-1β-deficient UB cells.

**Supplementary Table 4. Differentially expressed genes in HNF-1β-deficient UB cells.** RNA-seq was performed on UB cells purified from heterozygous control E14.5 kidneys and HNF-1β mutant kidneys. Log2(fold change) in expression between wild-type and HNF-1β deficient UB cells, FDR adjusted p-value, RNA-seq count data for each sample, and mean counts for wild-type and HNF-1β deficient samples are shown.

| Mark | Activated/Repressed by HNF-1β | Annotation | n | n_zero | q1 | q3 | median | mean | se |
| --- | --- | --- | --- | --- | --- | --- | --- | --- | --- |
| HNF-1β | Activated | Promoter | 239 | 0 | 2.03 | 2.48 | 2.20 | 2.27 | 0.02 |
| HNF-1β | Repressed | Promoter | 174 | 0 | 1.97 | 2.38 | 2.15 | 2.19 | 0.02 |
| ATAC | Activated | Promoter | 239 | 0 | 0.68 | 2.58 | 1.34 | 2.62 | 0.22 |
| ATAC | Repressed | Promoter | 174 | 0 | 0.57 | 3.53 | 1.41 | 2.90 | 0.28 |
| H3K27ac | Activated | Promoter | 239 | 0 | 0.85 | 2.18 | 1.43 | 1.84 | 0.11 |
| H3K27ac | Repressed | Promoter | 174 | 0 | 0.59 | 2.62 | 1.06 | 1.94 | 0.15 |
| H3K4me1 | Activated | Promoter | 239 | 0 | 0.71 | 1.26 | 0.97 | 1.01 | 0.03 |
| H3K4me1 | Repressed | Promoter | 174 | 0 | 0.64 | 1.23 | 0.87 | 0.97 | 0.04 |
| HNF-1β | Activated | Intragenic | 361 | 0 | 2.07 | 2.44 | 2.23 | 2.27 | 0.02 |
| HNF-1β | Repressed | Intragenic | 539 | 0 | 2.01 | 2.41 | 2.22 | 2.24 | 0.01 |
| ATAC | Activated | Intragenic | 361 | 0 | 0.38 | 1.42 | 0.73 | 1.08 | 0.06 |
| ATAC | Repressed | Intragenic | 539 | 0 | 0.25 | 0.96 | 0.45 | 0.75 | 0.04 |
| H3K27ac | Activated | Intragenic | 361 | 0 | 0.53 | 1.50 | 0.88 | 1.15 | 0.05 |
| H3K27ac | Repressed | Intragenic | 539 | 0 | 0.36 | 0.87 | 0.54 | 0.72 | 0.03 |
| H3K4me1 | Activated | Intragenic | 361 | 0 | 0.61 | 1.26 | 0.90 | 0.98 | 0.03 |
| H3K4me1 | Repressed | Intragenic | 539 | 0 | 0.39 | 0.97 | 0.63 | 0.74 | 0.02 |
| HNF-1β | Activated | Distal Intergenic | 139 | 0 | 2.01 | 2.46 | 2.23 | 2.28 | 0.03 |
| HNF-1β | Repressed | Distal Intergenic | 154 | 0 | 2.02 | 2.42 | 2.19 | 2.24 | 0.02 |
| ATAC | Activated | Distal Intergenic | 139 | 0 | 0.32 | 1.32 | 0.61 | 1.02 | 0.10 |
| ATAC | Repressed | Distal Intergenic | 154 | 0 | 0.26 | 0.80 | 0.43 | 0.81 | 0.12 |
| H3K27ac | Activated | Distal Intergenic | 139 | 0 | 0.40 | 1.21 | 0.66 | 1.03 | 0.09 |
| H3K27ac | Repressed | Distal Intergenic | 154 | 0 | 0.36 | 0.73 | 0.50 | 0.69 | 0.07 |
| H3K4me1 | Activated | Distal Intergenic | 139 | 0 | 0.43 | 0.99 | 0.69 | 0.77 | 0.04 |
| H3K4me1 | Repressed | Distal Intergenic | 154 | 0 | 0.36 | 0.86 | 0.55 | 0.66 | 0.03 |

| Mann-Whitney Wilcoxon Test | p-value |
| --- | --- |
| Intragenic HNF-1β | 2.55E-01 |
| Intragenic ATAC | 2.32E-09 |
| Intragenic H3K27ac | 2.56E-19 |
| Intragenic H3K4me1 | 3.91E-15 |
| Distal Intergenic HNF-1β | 3.49E-01 |
| Distal Intergenic ATAC | 1.46E-03 |
| Distal Intergenic H3K27ac | 3.55E-04 |
| Distal Intergenic H3K4me1 | 2.27E-02 |
| Promoter HNF-1β | 2.94E-02 |
| Promoter ATAC | 8.89E-01 |
| Promoter H3K27ac | 1.78E-01 |
| Promoter H3K4me1 | 1.10E-01 |

**Supplementary Table 5. Enrichment of ATAC-seq, H3K27ac, and H3K4me1 at HNF-1β binding sites.** Median and mean enrichment values of HNF-1β binding, ATAC-seq, H3K27 acetylation (H3K27ac), and H3K4 monomethylation (H3K4me1) in intragenic and distal intergenic regions of genes that are activated or repressed by HNF-1β are shown as RPKM (Reads Per Kilobase of sequence range per Million mapped reads). Mann-Whitney Wilcoxon tests were used to test whether the differences between activated and repressed genes were significant for each mark and region.

**Supplementary Table 6. HNF-1β binding sites with H3K27ac and H3K4me1 co-occupancy.** List of 1,577 HNF-1β binding sites in E14.5 kidneys that are within 1 kb of an H3K4me1 peak and within 1 kb of an H3K27ac peak. Genomic range, genomic region annotation, gene ID and name, and Log2fc in HNF-1β mutant UB cells versus heterozygous control are shown.

| Cell Type | n | n_zero | q1 | q3 | median | mean | se |  |
| --- | --- | --- | --- | --- | --- | --- | --- | --- |
| Immune | 1606 | 655 | 0.00 | 2.80 | 0.65 | 3.42 | 0.24 |  |
| NP | 1606 | 126 | 0.16 | 3.47 | 0.94 | 3.80 | 0.20 |  |
| PC | 1606 | 226 | 0.18 | 8.34 | 2.32 | 6.49 | 0.25 |  |
| IC | 1606 | 406 | 0.00 | 7.75 | 1.62 | 6.47 | 0.30 |  |
|  |  |  |  |  |  |  |  |  |
| Cell Type | Activated/Repressed by HNF-1β | n | n_zero | q1 | q3 | median | mean | se |
| IC | Activated by HNF-1β | 739 | 115 | 0.47 | 11.40 | 3.47 | 8.43 | 0.46 |
| IC | Repressed by HNF-1β | 867 | 291 | 0.00 | 4.86 | 0.66 | 4.81 | 0.38 |
| PC | Activated by HNF-1β | 739 | 63 | 0.55 | 12.39 | 4.37 | 8.74 | 0.42 |
| PC | Repressed by HNF-1β | 867 | 163 | 0.09 | 5.43 | 1.07 | 4.58 | 0.29 |
|  |  |  |  |  |  |  |  |  |
| Mann-Whitney Wilcoxon Test | p-value |  |  |  |  |  |  |  |
| Immune vs NP snATAC | 1.3E-22 |  |  |  |  |  |  |  |
| Immune vs PC snATAC | 1.29E-54 |  |  |  |  |  |  |  |
| Immune vs IC snATAC | 1.21E-29 |  |  |  |  |  |  |  |
| NP vs PC snATAC | 3.48E-13 |  |  |  |  |  |  |  |
| NP vs IC snATAC | 0.002289 |  |  |  |  |  |  |  |
| PC Activated vs Repressed snATAC | 8.46E-26 |  |  |  |  |  |  |  |
| IC Activated vs Repressed snATAC | 3.61E-24 |  |  |  |  |  |  |  |

**Supplementary Table 7. Enrichment of principal cell and intercalated cell snATAC-seq at HNF-1β binding sites.** Median and mean enrichment values of snATAC-seq (single nuclear ATAC-seq) at HNF-1β binding sites in immune cells, nephron progenitors (NP), principal cells (PC), and intercalated cells (IC) are shown as RPKM (Reads Per Kilobase of sequence range per Million mapped reads). The Mann-Whitney Wilcoxon Test was used to test the significance of differences in enrichment.

| **Canonical Pathways** | **-log(p-value)** | **Ratio** | **Genes** |
| --- | --- | --- | --- |
| Hepatic Fibrosis / Hepatic Stellate Cell Activation | 11.60 | 0.240 | FGFR2,IGFBP4,FGFR1,IL1RL1,FGF1,MMP9,MMP2,COL6A1,PGF,IL6R,COL1A1,IFNGR1,COL12A1,COL9A3,LY96,COL20A1,ECE1,EDN1,TGFB3,PDGFRB,COL8A2,COL6A3,LAMA1,TNFRSF1B,VEGFA,CD14,PDGFRA,COL3A1,TGFB2,COL1A2,MYL9,COL6A2,COL5A1,TIMP2,PDGFC,COL4A6,BCL2,IL18RAP,COL2A1,TIMP1,COL24A1,COL11A2,FAS,ICAM1 |
| Axonal Guidance Signaling | 10.90 | 0.171 | BMP5,MMP2,TUBB3,ADAMTS2,SEMA4A,FZD5,ENPEP,TUBA1A,BMP4,SEMA3C,UNC5B,FZD6,ADAM12,WNT6,BDNF,EFNB2,PIK3R3,EPHB3,VEGFA,PLCB4,NGEF,SEMA6A,ADAM19,MYL9,SRGAP1,SEMA3D,FZD4,PDGFC,EPHA1,SHANK2,GNG12,TUBA4A,NTRK1,BMP3,SEMA4D,GNAI1,ARHGEF15,GNB4,MMP9,WNT5A,NRP2,WNT10A,PAPPA,ITGA2,FZD2,PGF,SEMA3F,PLXNC1,MICAL1,PTCH2,TUBB2A,ADAMTS6,FYN,ADAMTS9,PLCL1,KALRN,BMP7,PLCB1,NFATC4,SEMA6B,ADAMTS4,SDC2,PAK1,SEMA3B,WNT5B,ECEL1,RASSF5,PIK3CG,UNC5C,SEMA3G,MYLPF,SEMA3A,ADAMTS7,PRKCG |
| LPS/IL-1 Mediated Inhibition of RXR Function | 7.39 | 0.186 | IL1RL1,CPT1A,PPARA,MGST2,NR5A2,ACSL6,ALDH1A2,LY96,PPARGC1A,HS3ST5,GSTM2,FABP5,CHST1,IL33,CHST2,CHST3,GSTA3,ALDH1L1,NDST3,FABP4,TNFRSF1B,CD14,NR1H4,ABCG5,ABCA1,SLCO1A2,Sult1d1,ABCG8,UST,CHST11,ALDH4A1,SULT1C2,IL18,MAP3K1,IL18RAP,ALDH5A1,ACSBG1,GSTM4,HS3ST1,ALDH1A3,CHST15 |
| Regulation of the Epithelial-Mesenchymal Transition Pathway | 7.12 | 0.196 | FGFR2,CDH2,LOX,FGFR1,FGF1,MMP9,MMP2,WNT5A,WNT10A,FZD2,ZEB2,FZD5,SNAI1,SNAI2,PARD6B,FGF13,FZD6,CDH1,WNT6,FGFR4,TGFB3,PDGFRB,PIK3R3,NOTCH1,JAG1,LEF1,WNT5B,FGF7,TGFB2,NOTCH4,JAG2,FZD4,PIK3CG,ESRP2,TWIST2,HNF1A |
| Human Embryonic Stem Cell Pluripotency | 6.29 | 0.209 | FGFR2,FGFR1,BMP5,WNT5A,WNT10A,FZD2,NOG,FZD5,BMP4,FZD6,WNT6,FGFR4,BDNF,BMP7,S1PR1,TGFB3,PDGFRB,PIK3R3,PDGFRA,LEF1,WNT5B,TGFB2,FZD4,PIK3CG,PDGFC,HNF1A,NTRK1,BMP3 |
| Role of Osteoblasts, Osteoclasts and Chondrocytes in Rheumatoid Arthritis | 5.66 | 0.169 | IL1RL1,BMP5,DKK3,WNT5A,WNT10A,ITGA2,FZD2,COL1A1,CSF1R,FZD5,FOS,BMP4,FZD6,LRP1,WIF1,WNT6,SPP1,IL33,BMP7,NFATC4,ADAMTS4,MAP3K5,PIK3R3,TNFRSF1B,LEF1,WNT5B,TRAF5,FZD4,PIK3CG,SMAD9,ALPL,IL18,PTK2B,BCL2,IL18RAP,HNF1A,BMP3 |
| Colorectal Cancer Metastasis Signaling | 5.28 | 0.161 | MMP25,GNB4,MMP9,MMP2,WNT5A,WNT10A,TLR1,FZD2,PGF,IL6R,FZD5,ADCY1,FOS,IFNGR1,FZD6,LRP1,CDH1,WNT6,TLR6,MMP28,RND3,ADCY4,TGFB3,PIK3R3,ADCY7,VEGFA,LEF1,WNT5B,TGFB2,PTGER4,FZD4,PIK3CG,PDGFC,NOS2,MMP19,GNG12,HNF1A,MMP16 |
| Granulocyte Adhesion and Diapedesis | 5.17 | 0.175 | GNAI1,Ccl2,MMP25,IL1RL1,MMP9,MMP2,ITGA2,CCL20,HRH1,CLDN1,CLDN2,CX3CL1,Cxcl11,ITGA1,CCL17,CLDN20,MMP28,SDC3,IL33,Ccl7,TNFRSF1B,SDC2,ITGA6,IL18,MMP19,CLDN9,IL18RAP,JAM3,ICAM1,PECAM1,MMP16 |
| FXR/RXR Activation | 4.29 | 0.183 | GC,FOXA3,SERPINF1,PKLR,VLDLR,NR1H4,ABCG5,PPARA,ABCG8,NR5A2,PPARG,IL18,C4A/C4B,PPARGC1A,CLU,KNG1,FETUB,HNF1A,LCAT,FGFR4,AMBP,IL33,G6PC2 |
| Dermatan Sulfate Biosynthesis (Late Stages) | 4.10 | 0.267 | CHST2,CHST3,DSE,NDST3,HS3ST5,Sult1d1,HS3ST1,CHST1,UST,CHST11,SULT1C2,CHST15 |
| Inhibition of Matrix Metalloproteases | 4.05 | 0.282 | MMP25,MMP9,MMP19,MMP2,SDC2,ADAM12,LRP1,TIMP1,MMP28,TIMP2,MMP16 |
| Chondroitin Sulfate Biosynthesis (Late Stages) | 3.90 | 0.255 | CHST2,CHST3,NDST3,HS3ST5,CHSY3,Sult1d1,HS3ST1,CHST1,UST,CHST11,SULT1C2,CHST15 |
| Leukocyte Extravasation Signaling | 3.81 | 0.152 | GNAI1,MMP25,MMP9,MMP2,ITGA2,CLDN1,CLDN2,NCF2,ITGA1,DLC1,CLDN20,MMP28,SPN,PIK3R3,ITGA6,RASSF5,TIMP2,PIK3CG,MMP19,PTK2B,CLDN9,MAPK13,JAM3,TIMP1,ICAM1,Actn3,PECAM1,ARHGAP9,MMP16,PRKCG |
| Agranulocyte Adhesion and Diapedesis | 3.80 | 0.153 | GNAI1,Ccl2,MMP25,MMP9,MMP2,ITGA2,CCL20,HRH1,CLDN1,Podxl,CLDN2,CX3CL1,Cxcl11,ITGA1,CCL17,CLDN20,MMP28,IL33,Ccl7,ITGA6,MYL9,IL18,MMP19,CLDN9,AOC3,JAM3,ICAM1,PECAM1,MMP16 |
| Role of Macrophages, Fibroblasts and Endothelial Cells in Rheumatoid Arthritis | 3.74 | 0.135 | CAMK2B,IL1RL1,DKK3,WNT5A,SOCS3,WNT10A,TLR1,FZD2,PGF,IL6R,FZD5,FOS,FZD6,LRP1,WIF1,WNT6,TLR6,PLCL1,IL33,PLCB1,NFATC4,ADAMTS4,PIK3R3,TNFRSF1B,VEGFA,LEF1,PLCB4,LTA,WNT5B,TRAF5,FZD4,PIK3CG,PDGFC,IL18,NOS2,IL18RAP,HNF1A,SOCS1,ICAM1,PRKCG |
| LXR/RXR Activation | 3.65 | 0.174 | GC,IL1RL1,MMP9,TNFRSF1B,CD14,SERPINF1,NR1H4,ABCG5,ABCA1,ABCG8,LYZ,LY96,IL18,C4A/C4B,NOS2,CLU,IL18RAP,KNG1,LCAT,AMBP,IL33 |
| Basal Cell Carcinoma Signaling | 3.64 | 0.208 | BMP5,WNT5A,WNT10A,LEF1,FZD2,WNT5B,PTCH2,FZD4,FZD5,BMP4,HNF1A,FZD6,WNT6,BMP7,BMP3 |
| Xenobiotic Metabolism Signaling | 3.56 | 0.136 | CAMK2B,MGST2,NQO1,ALDH1A2,PPARGC1A,HS3ST5,GSTM2,CHST1,CYP1B1,NRIP1,CHST2,CHST3,GCLC,GSTA3,MAP3K5,PIK3R3,ALDH1L1,NDST3,UGT2B10,Ugt1a7c,CAMK1D,Sult1d1,MAF,UST,CHST11,ALDH4A1,SULT1C2,PIK3CG,NOS2,MAP3K1,ALDH5A1,MAPK13,GSTM4,HS3ST1,ALDH1A3,PRKCG,CHST15 |
| Dermatan Sulfate Biosynthesis | 3.56 | 0.224 | CHST2,CHST3,DSE,NDST3,CHSY3,Sult1d1,UST,CHST11,SULT1C2,HS3ST5,HS3ST1,CHST1,CHST15 |
| Hepatic Cholestasis | 3.44 | 0.155 | IL1RL1,CYP7B1,PPARA,NR5A2,ADCY1,LY96,FGFR4,ESR1,IL33,ADCY4,TGFB3,ADCY7,TNFRSF1B,CD14,NR1H4,ABCG5,LTA,SLCO1A2,TGFB2,LIF,ABCG8,IL18,IL18RAP,HNF1A,PRKCG |
| Factors Promoting Cardiogenesis in Vertebrates | 3.39 | 0.185 | TGFB3,BMP5,LEF1,FZD2,TGFB2,NOX4,NOG,FZD4,FZD5,SMAD9,BMP4,HNF1A,FZD6,LRP1,BMP7,BMP3,PRKCG |
| Sphingosine-1-phosphate Signaling | 3.38 | 0.174 | PLCB1,GNAI1,ADCY4,PDGFRB,PIK3R3,SMPD3,ADCY7,PDGFRA,PLCB4,CASP1,CASQ1,PIK3CG,PDGFC,ADCY1,PTK2B,CASP4,RND3,PLCL1,S1PR1 |
| HIF1α Signaling | 3.30 | 0.176 | MMP25,PIK3R3,MMP9,SLC2A3,MMP2,VEGFA,EGLN3,SLC2A2,PGF,PIK3CG,PDGFC,NOS2,MMP19,MAPK13,EDN1,MMP28,MMP16,LDHB |
| Maturity Onset Diabetes of Young (MODY) Signaling | 3.24 | 0.333 | GCK,PKLR,SLC2A2,CACNA1C,HNF1A,HNF1B,PDX1 |
| Molecular Mechanisms of Cancer | 3.23 | 0.123 | CAMK2B,GNAI1,ARHGEF15,BMP5,WNT5A,WNT10A,ITGA2,FZD2,IRS1,PTCH2,FZD5,ADCY1,FOS,FYN,ARHGEF16,BMP4,CDKN1A,FZD6,LRP1,CDH1,WNT6,ARHGEF4,RND3,BMP7,PLCB1,ADCY4,TGFB3,MAP3K5,PIK3R3,NOTCH1,ADCY7,LEF1,PLCB4,PAK1,WNT5B,TGFB2,FZD4,PIK3CG,SMAD9,GAB2,BCL2,MAPK13,FAS,BMP3,PRKCG |

**Supplementary Table 8. Axon guidance is a highly enriched pathway in HNF-1β-deficient mIMCD3 cells.** Ingenuity pathway analysis was performed on 1,894 differentially-expressed genes with log_2_ fold-change >1 or <-1 in HNF-1β-deficient mIMCD3 cells compared to wild-type cells. Table shows -log(p-value), ratio of differentially-expressed genes to total genes, and list of differentially-expressed genes. The 25 most significantly enriched pathways are shown.
